# Silmitasertib (CX-4945), a Clinically Used CK2-Kinase Inhibitor with Additional Effects on GSK3β and DYRK1A Kinases: A Structural Perspective

**DOI:** 10.1021/acs.jmedchem.2c01887

**Published:** 2023-03-08

**Authors:** Przemyslaw Grygier, Katarzyna Pustelny, Jakub Nowak, Przemyslaw Golik, Grzegorz M. Popowicz, Oliver Plettenburg, Grzegorz Dubin, Filipe Menezes, Anna Czarna

**Affiliations:** † Malopolska Centre of Biotechnology, Jagiellonian University, Gronostajowa 7A, 30-387 Krakow, Poland; ‡ Selvita S.A, Bobrzynskiego, 14, 30-338 Krakow, Poland; § Institute of Structural Biology, Helmholtz Zentrum Muenchen, Ingolstaedter Landstrasse 1, Neuherberg 85764, Germany; ∥ Biomolecular NMR and Center for Integrated Protein Science Munich at Department Chemie, Technical University of Munich, Lichtenbergstrasse 4, Garching 85747, Germany; ⊥ Institute of Medicinal Chemistry, Helmholtz Munich, Ingolstaedter Landstrasse 1, Neuherberg 85764, Germany; # Institute of Organic Chemistry, Centre of Biomolecular Drug Research (BMWZ) and Laboratory of Nano and Quantum Engineering (LNQE), Leibniz University Hannover, Schneiderberg 1b, Hannover 30167, Germany; ∇ German Center for Diabetes Research (DZD), Ingolstaedter Landstrasse 1, Neuherberg 85764, Germany; ○ Institute of Lung Health (ILH), Aulweg 130, Giessen 35392, Germany

## Abstract

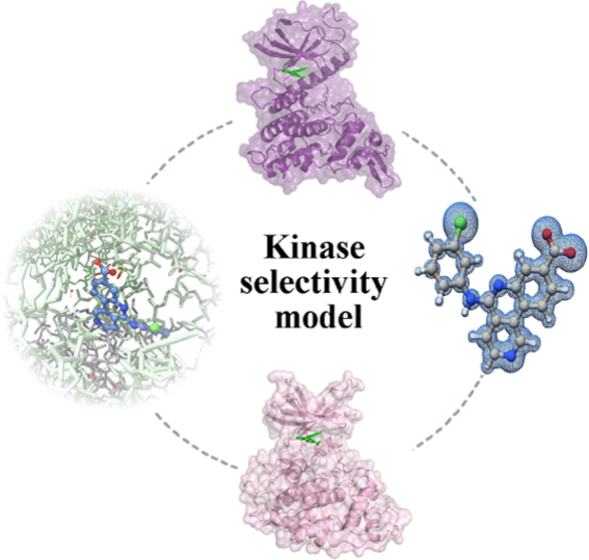

A clinical casein kinase 2 inhibitor, CX-4945 (silmitasertib), shows significant affinity toward the DYRK1A and GSK3β kinases, involved in down syndrome phenotypes, Alzheimer’s disease, circadian clock regulation, and diabetes. This off-target activity offers an opportunity for studying the effect of the DYRK1A/GSK3β kinase system in disease biology and possible line extension. Motivated by the dual inhibition of these kinases, we solved and analyzed the crystal structures of DYRK1A and GSK3β with CX-4945. We built a quantum-chemistry-based model to rationalize the compound affinity for CK2α, DYRK1A, and GSK3β kinases. Our calculations identified a key element for CK2α’s subnanomolar affinity to CX-4945. The methodology is expandable to other kinase selectivity modeling. We show that the inhibitor limits DYRK1A- and GSK3β-mediated cyclin D1 phosphorylation and reduces kinase-mediated NFAT signaling in the cell. Given the CX-4945’s clinical and pharmacological profile, this inhibitory activity makes it an interesting candidate with potential for application in additional disease areas.

## Introduction

The human kinome contains 518 protein kinases accounting for 1.7% of all human genes.^1^ Kinases control a variety of physiological processes, including cell growth, differentiation, proliferation, angiogenesis, apoptosis, cytoskeleton rearrangement, metabolism, and others.^2^ Deregulation of specific kinases has been linked to virtually all major disease areas.^3^ Consequently, kinases became one of the most important targets for drug discovery,^4^ with 73 kinase inhibitor drugs authorized to date by the FDA.^5^

CX-4945 (silmitasertib) is the first orally bioavailable inhibitor of casein kinase 2 (CK2) with acceptable pharmacological properties.^6^ CX-4945 is a molecule with a relatively low polar surface area, few rotatable bonds, and low aqueous solubility, bearing one carboxylic acid and two weakly basic aromatic nitrogen residues. Its molecular properties are summarized in the Supporting Material, Table S1. It was granted orphan drug designation by the FDA for cholangiocarcinoma (bile duct cancer) in 2017 and is developed as a drug candidate in other solid tumors.^7^ Phosphorylation of cellular targets by CK2 was reported to facilitate SARS-CoV-2 spread, and subsequently, antiviral activity for CX-4945 was reported.^8^ Clinical trials to demonstrate putative benefits are ongoing.^9^ CX-4945 is an ATP-competitive inhibitor characterized by a K_i_ of 380 pM for CK2α. Although it inhibits a few off-target kinases (*e.g.*, PIM1, HIPK3, or CLK3),^10^ the reported adverse effects are moderate. This illustrates a current paradigm shift, where targeting multiple kinases with related substrate phosphorylation patterns is considered advantageous over highly specific kinase inhibitors.^11−13^

The dual-specificity tyrosine phosphorylation-regulated kinase 1A (DYRK1A) encoding gene is located in the Down syndrome critical region and was originally associated with neurodegenerative diseases, including Alzheimer’s and Down syndrome.^14^ More recent findings also implicated the role of DYRK1A in cancer.^15,16^ DYRK1A phosphorylation primes the substrates for subsequent phosphorylation by a processive kinase, the constitutively active glycogen synthase kinase-3β (GSK3β).^17,18^ GSK3β phosphorylates more than a hundred different substrates^19^ and has been implicated in diverse cellular processes, including embryogenesis, immune response, inflammation, apoptosis, autophagy, wound healing, neurodegeneration, and carcinogenesis.^20^ Abnormal regulation of GSK3β has been linked to the onset and progression of chronic conditions, including cancer, diabetes, neurodegenerative, and behavioral diseases.^21^

The processive kinase GSK3β and its priming kinase DYRK1A are critical for the regulation of β-cell function. The inhibition of DYRK1A and GSK3β brings a synergistic effect, leading to an increase in insulin release and β-cell proliferation. The synergism has been attributed, among others, to the regulation of ion channels responsible for the exocytosis of insulin from storage granules, and its benefits on β cell health could already impressively be demonstrated by an aminopyrazine series of inhibitors.^22^ The latter possess about equipotent DYRK1A and GSK3β inhibitory efficacy. This improvement of glucose homeostasis by the synergistic inhibition of DYRK1A and GSK3β hints at an opportunity for the curative therapy of diabetes.^23−25^

DYRK1A and GSK3β are serine–threonine kinases of the CMGC family and share many structural features.^26^ This opens the possibility for developing dual inhibitors, which is especially attractive since the latter may benefit from the synergistic effects mentioned above. Many ATP-competitive inhibitors and allosteric modulators of GSK3β have been reported.^27^ Several DYRK1A inhibitors have also been discovered and investigated in the context of neurodegenerative disorders^28,29^ and autism.^30^ While inhibiting multiple CMGC kinases,^31^ the inhibitors reported to date are characterized by relatively poor affinities. Prior studies have demonstrated CX-4945’s inhibition of DYRK1A,^32^ prompting us to evaluate its utility as an antidiabetic. As silmitasertib is already undergoing clinical evaluation in patients, its overall safety profile seemed promising. Furthermore, the reported primary activity as a potent CK2 inhibitor can possibly exert additional beneficial effects in diabetes,^33^ thus further increasing the proposed synergism.

Here, we present the results of a structural and computational evaluation of CX-4945 as a dual DYRK1A and GSK3β kinase inhibitor. The crystal structures of the inhibitor in complex with DYRK1A and both the phosphorylated and non-phosphorylated forms of GSK3β kinase reveal the binding mode of CX-4945 in those kinases. Rational justification of CX-4945’s binding and inhibitory power from the crystal structures required the involvement of quantum chemical calculations. This allowed the identification and assignment of crucial contributions and roles for each functional group of the inhibitor for binding. This method, which translates structural data into different energy contributions, can be extrapolated to other kinases. Moreover, we show functional implications of the inhibition of DYRK1A and GSK3β at the cellular levels.

## Results

### CX-4945 is a Potent Inhibitor of DYRK1A and GSK3β

**Figure 1:**
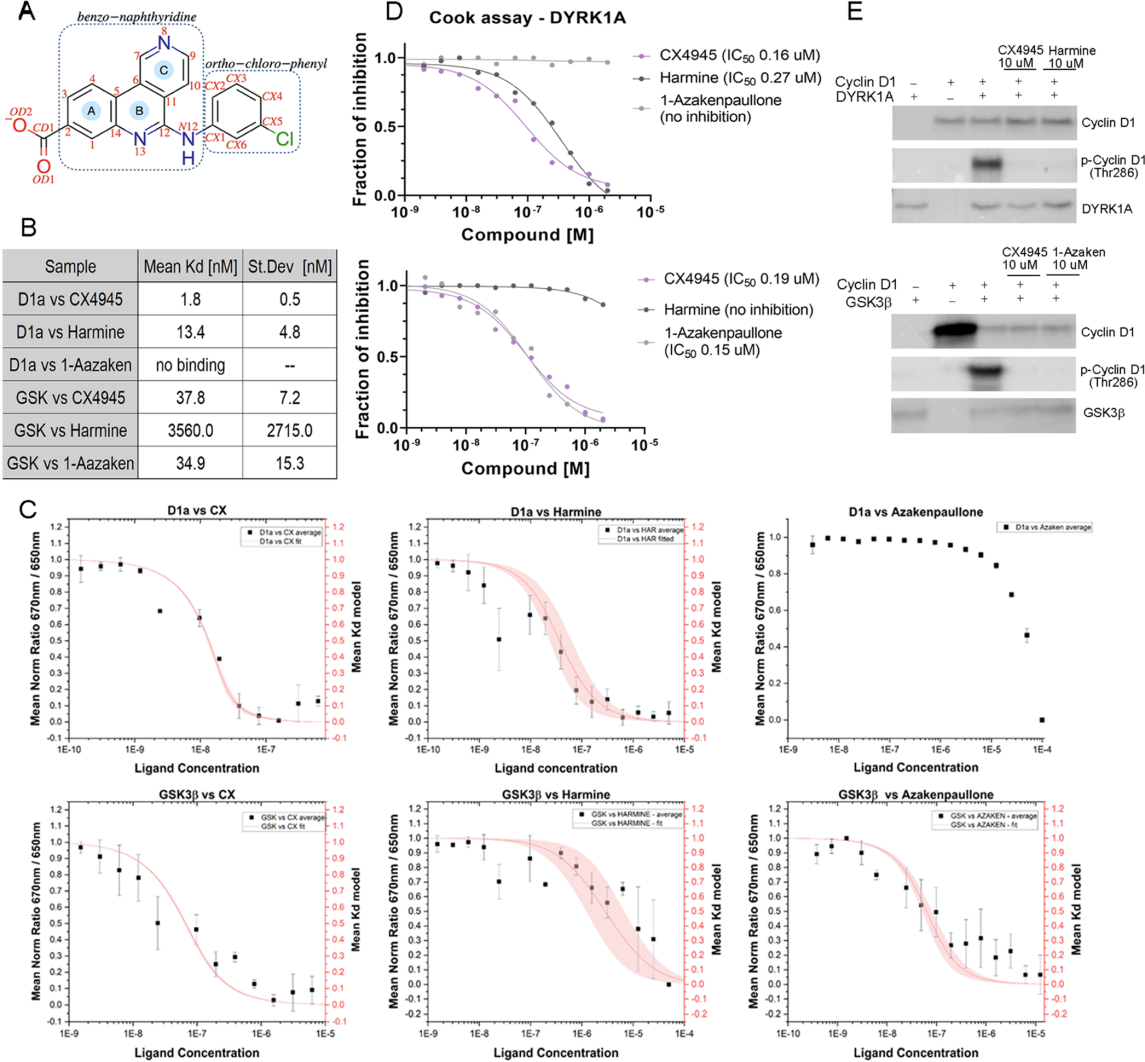
(A) Chemical structure of CX-4945 (silmitasertib). (B, C) Direct interaction determined by microscale thermophoresis (MST) of an inhibitor with DYRK1A (upper panel) and GSK3β (lower panel), with the *K*_d_ value summarized in the table. (D) Inhibitory activity of CX-4945 against DRYRK1A (upper panel) and GSK3β (lower panel) as determined in the Cook kinase activity assay. (E) CX-4945 inhibits DYRK1A and GSK3β-mediated phosphorylation of Cyclin D1 in the transiently transfected HEK293T cells with plasmids encoding HA-Cyclin D1 and FLAG-DYRK1A or FLAG-GSK3β. The protein profile was analyzed in cell lysate with Western Blot using specific monoclonal antibodies anti-FLAG (DYRK1A and GSK3β), anti-HA (Cyclin D1), and anti-phosho-Cyclin D1.

CX-4945 (Figure 1A) is a potent ATP-competitive small molecule inhibitor of CK2, with the unusual structural feature of having a free carboxylic acid. This is rarely seen in kinase inhibitors. For targeting CK2α, however, this functional group seems to be beneficial, as it is a characteristic shared with other inhibitors, like TTP22 or CX-5011. CX-4945 also inhibits kinases from the CMGC family, including the dual-specificity protein kinases CLK2, CLK3, and serine/threonine homeodomain-interacting protein kinase 3 (HIPK3) or others.^34,35^ Additionally, CX-4945 was reported earlier to block DYRK1A.^32^ In our effort to identify dual DYRK1A/GSK3β inhibitors, we decided to evaluate the efficacy of CX-4945 as a potential multitarget inhibitor.

First, we investigated the direct interaction of three compounds with DYRK1A and GSK3β using microscale thermophoresis (MST). Our tests included CX-4945, harmine, a known inhibitor of DYRK1A,^36^ and 1-azakenpaullone, an inhibitor of GSK3β.^37^ The recombinant kinase domains of DYRK1A (126–490) and GSK3β (26–383), both with C-terminal His-tag extension, were expressed in *E. coli*, purified to homogeneity, and labeled with a relevant fluorescent dye (*cf.*Experimental Section). Then, the dissociation constants were determined in the direct binding assay using constant concentrations of DYRK1A or GSK3β kinase domains (20 and 62.5 nM, respectively) titrated with increasing concentrations of the compounds. Increasing the concentration of the small molecules dose-dependently affected the thermophoretic profile of both DYRK1A and GSK3β, suggesting physical interaction with both kinases (Figure 1C). Expectedly, harmine was significantly more effective toward DYRK1A than GSK3β. On the other hand, 1-azakenpaullone affected only its known target, GSK3β, but not DYRK1A, demonstrating the specificity of the assay. Our results indicate that CX-4945 strongly binds to both kinases with nanomolar dissociation constants (*K*_d_) (Figure 1B). The affinity of CX-4945 toward DYRK1A was higher compared to the reference compound harmine (*K*_d_ 1.8 and 13.4 nM, respectively), while for GSK3β it was comparable to that of the reference compound, 1-azakenpaullone (37.8 nM).

In a global analysis of 243 clinically evaluated kinase drugs,^38^ the reported *K*_d,app_ (apparent) of DYRK1A-CX-4945 and GSK3β-CX-4945 were 35 and 4800 nM, respectively. This K_d,app_ was calculated based on EC_50_ values from the Kinobeads assay of the cell lysates titrated with compounds. The values obtained were 35.2 ± 20.74 nM for DYRK1A and 10 429.37 ± 32 287.91 nM for GSK3β (Table S2 of ref (38)). The observed discrepancy may be due to the nature of the proteins analyzed: in our MST assay, truncated kinases (active kinase domains) were used, while in the Kinobeads assay, the whole proteins were analyzed. Moreover, the activation status of an endogenous kinase in cells may not be the same as that of a recombinant protein in a cell-free system. Nevertheless, both assays identified DYRK1A and GSK3β as CX-4945 targets.

Next, we checked whether CX-4945 was able to impair DYRK1A- and GSK3β-mediated phosphorylation. The inhibitory potency (IC_50_) was evaluated using the Cook activity assay.^39^ For this, a fixed amount of the kinase (0.25 μM) was titrated with the tested inhibitors (1 nM to 10 μM) in the presence of ATP (128 μM) and a substrate peptide (0.5 mM). The ATP concentration was selected based on the experimentally determined *K*_M_ of 118 and 128 μM for DYRK1A and GSK3β, respectively. The DYRKtide peptide (RRRFRPASPLRGPPK) was used as a substrate for DYRK1A, while the GYS1 peptide (YRRAAVPPSPSLSRHSSPHQ(pS)EDEEE) with the phosphoSer residue in the +4 position was used for GSK3β. CX-4945 turned out to be a very potent inhibitor of both kinases with potency (IC_50_) in the nanomolar range (Figure 1D). For DYRK1A, once more, CX-4549 proved to be more active than harmine (IC_50_ of 0.16 and 0.27 μM, respectively) (Figure 1D upper panel), while no inhibitory activity was detected for 1-azakenpaullone. Our results from the Cook assay correlate well with the previously published data for DYRK1A,^32^ where CX-4945 inhibits this kinase in the nanomolar range and with higher potency than harmine. The small discrepancies observed are due to assay sensitivity. CX-4945 reveals furthermore similar inhibitory activity against other kinases from the DYRK family, like DYRK2.^10^ Moreover, CX-4945 strongly inhibits GSK3β with IC_50_ values of 0.19 μM and shows an inhibitory power comparable to 1-azakenpaullone (IC_50_ 0.15 μM) (Figure 1D lower panel). In the kinome scan with a single point inhibition readout presented by Battistutta and co-workers, CX-4945 showed 55% inhibition of GSK3β at 500 nM,^10^ while in our assay, with a concentration range of 1 nM to 10 μM, we observed 50% inhibition at 190 nM. Our CX-4945 values are slightly shifted toward higher activity.

To further confirm our findings, we analyzed the effect of CX-4945 binding on the thermal stability of both kinases. The protein’s melting temperatures in the presence of the inhibitors were determined using the dye-based thermal-shift assay (Figure S3).^40^ The thermal-shift assay indicated that binding of CX-4945 significantly stabilized DYRK1A and GSK3β kinase domains and induced shifts of 12 °C and 9.5 °C, respectively. For DYRK1A, the observed stabilizing effect of CX-4945 was, once more, stronger than that of harmine (10.5 °C), while 1-azakenpaullone induced only slight changes in the protein’s melting temperature (2 °C). For GSK3β, the interaction with 1-azakenpaullone had a more prominent effect (13 °C), but no temperature stabilization effect was observed after incubation with harmine.

Our data establish CX-4945 as a dual DYRK1A and GSK3β inhibitor with *in vitro* potency allowing us to expect a biological effect. To evaluate our hypothesis, we tested whether CX-4945 would inhibit DYRK1A and GSK3β in mammalian cell lines. GSK3β is a constitutively active kinase whose activity in a cell is modulated by various kinases through phosphorylation at Ser9. Therefore, we used only GSK3β (26–383) kinase domain constructs in our cellular studies. Consequently, the observed effects are caused by the inhibitors and not by other cellular events. Among the common substrates of both kinases, we selected Cyclin D1, which is a positive regulator of the cell cycle. It controls the transition from a proliferative to a quiescent state and determines the fate of the cell. Both DYRK1A and GSK3β were shown to be required for Cyclin D1 phosphorylation. This, in turn, leads to the nuclear export of Cyclin D1 and its subsequent degradation in the proteasome.^41,42^ Moreover, Cyclin D1 belongs to a limited group of GSK3β substrates for which priming is not mandatory,^43^ and thus allows the direct investigation of GSK3β inhibition. The effect of CX-4945 on Cyclin D1’s phosphorylation status was evaluated in HEK293T cells, where the tested kinases and Cyclin D1 were transiently overexpressed. The protein profile was analyzed in cell lysate with Western Blot (Figure 1E) using specific monoclonal antibody anti-FLAG (DYRK1A and GSK3β), anti-HA (Cyclin D1), and anti-phospho-Cyclin D1 to exclude endogenous proteins. Thr289 was chosen because it is a known phosphorylation site of both tested kinases but not for Cyclin D-dependent kinases.^44^ When DYRK1A or GSK3β alone was overexpressed, the signal from phosphorylated Cyclin D1 was not detected, demonstrating that the endogenous expression of Cyclin D1 does not interfere with the assay’s results. Additionally, when Cyclin D1 alone was overexpressed, the amount of the endogenous phosphorylated form was negligible. The effect of DYRK1A overexpression on the phosphorylation of Cyclin D1 was fully abolished in the presence of harmine. The same effect was obtained with 1-azakenpaullone when GSK3β was overexpressed together with Cyclin D1. This demonstrates that the observed phosphorylation is indeed mediated by the respective kinases. Furthermore, for both kinases, the treatment with CX-4945 strongly inhibits Cyclin D1 phosphorylation, and complete inhibition was observed for 10 μM concentration.

Collectively, these results clearly demonstrate that CX-4945 is a potent DYRK1A and GSK3β inhibitor *in vitro* and in mammalian cells.

### Structural Basis of DYRK1A Inhibition by CX-4945: Binding to the ATP Pocket

To determine the molecular mechanism of DYRK1A inhibition by CX-4945, we solved the crystal structure of the kinase inhibitor complex at 2.77 Å resolution. The complex was crystallized in the C121 space group, with eight protein molecules found in the asymmetric unit. For all molecules in the asymmetric unit, the entire protein was well-ordered and comprised a long hairpin-like structure of an N-terminal DH box followed by a catalytic domain in the active kinase conformation (Figure S1A). Mass spectrometry analysis revealed heterogenous phosphorylation of our DYRK1A preparation (Figure S1C). However, the electron density maps showed only phosphorylation of DYRK1A at Tyr321, the second tyrosine of the YxY motif of the activation loop. This suggests that other phosphorylation spots are either of low frequency or located in regions undefined by the electron density.

**Figure 2:**
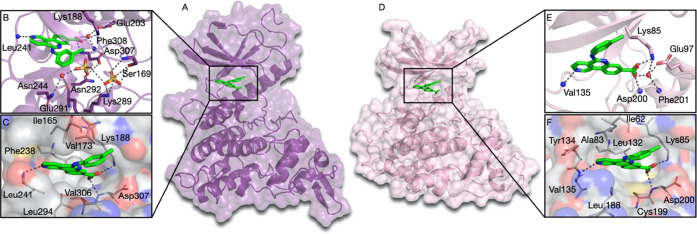
Crystal structure of CX-4945 bound to the active sites of DYRK1A and GSK3β. (A) The overall fold of DYRK1A (violet purple) in cartoon representation with CX-4945 (green sticks) at the ATP-binding pocket. (B) The insert showing CX-4945-DYRK1A interaction in the ATP-binding pocket. (C) Hydrophobic interactions stabilizing CX-4945 at the DYRK1A ATP-binding pocket. (D) Overview of the crystal structure of GSK3β (pink)–CX-4945 (green) complex. (E) Hydrogen-bond interactions of the inhibitor bound to the ATP pocket of GSK3β. (F) Hydrophobic interactions of CX-4945 at the ATP-binding pocket of GSK3β.

The electron density clearly defines the inhibitor in all eight protein–ligand complexes contained in the asymmetric unit (Figure S2A–H). The inhibitor-containing molecules superimpose with an average root-mean-square deviation (RMSD) below 0.4 Å over 320 C_α_ atoms. Because the inhibitor’s binding mode is equivalent in all complexes in the asymmetric unit, further discussion relates to molecule A unless indicated otherwise. CX-4945 occupies the ATP-binding site, sandwiched between the N- and C-lobes of the kinase domain (Figure 2A). In analogy to the protein–ligand structure docked by Kim and co-workers,^32^ the inhibitor is stabilized by hydrogen bonds involving functional groups in opposing parts of the benzo-naphthyridine moiety. Furthermore, we note that this is a well-conserved network of interactions among several kinases (Figure 5). In their work, Kim and colleagues mention the formation of 4 hydrogen bonds between the ligand and the protein, which slightly contrasts with our structure, where we count 3 instead. We attribute this difference to solvation waters, which seem to have been excluded from the docking strategy. On the other hand, our crystal structure improves on the relative orientation of the chlorophenyl group inside the pocket and lipophilic contacts, which were not entirely well captured in the docked structure. Besides the hydrogen-bond network and lipophilic interactions, water-mediated contacts are visible in our crystal model. The carboxyl moiety of the inhibitor contributes with a direct hydrogen bond with the main chain amide of Asp307 from the DFG motif (Figure 2B). Calculations based on model systems built from the crystal structure show that this is the strongest ligand–residue interaction with a direct hydrogen bond, amounting to −13.5 kcal/mol of stabilization. The interaction with the side chain of Lys188 is also particularly strong, and this is due to the coupled effect of a hydrogen bond with an ionic bridge between the two groups. Our calculations estimate that in the ligand–lysin contact, the ionic bridge amounts to 48% of the −12.6 kcal/mol interaction energy. The hydrogen bond for that same pair corresponds to 41.5%, and 10.5% is the interaction between the ligand and the alkyl chain of Lys188. Nearby carboxylates should decrease the strength of the interaction. Nonetheless, the synergy of Lys188 with Asp307 is a key element for binding, as it was previously used to anchor other DYRK1A inhibitors. This includes harmine, a potent and specific inhibitor of DYRK1A (PDB 3ANR).^45^ The carboxylate group of CX-4945 may additionally participate in a hydrogen bond with the main chain amide of Phe308 and a water-mediated contact with the side chain of Glu203 (Figure 2B). However, we estimate both interactions to be rather weak. In the latter, the carboxylate groups yield too much electrostatic repulsion (stabilization of −1.2 kcal/mol). Furthermore, note that the water-mediated bridge is not present in all complexes, further strengthening the observations from our calculations. For the interaction with Phe308, the large distance to the proton should be the main factor determining an enthalpic gain below 1 kcal/mol.

One of the nitrogen atoms on the benzo-naphthyridine fragment of the inhibitor (atom 8) contributes with a hydrogen bond with the main chain amide of Leu241 within the hinge region. Calculations on the isolated CX-4945-Leu241 fragment yield the estimate of −9.6 kcal/mol for this contact. The hydrogen bond alone is responsible for as much as 53% of the interaction energy in this ligand–residue pair. The adjacent main chain carboxyl oxygens of Glu239, Leu241, and Ser242 are involved in π-stacking interactions with the benzo-naphthyridine moiety. The latter stabilizes the complex with about −6.5 kcal/mol, and Glu239 is expected to be the main contributor. Hydrophobic interactions involve the benzo-naphthyridine and the side chains of Ile165, Ala186, Val173, Val222, Val306, and Phe238 from the N-lobe, Met240 of the hinge region, and Leu294 and Val306 from the C-lobe (Figure 2C). Model systems based solely on all of the side chains of those amino acids resulted in a contribution of −14.8 kcal/mol to the binding energy. The chlorine atom of the chlorophenyl moiety of CX-4945 resides in a shallow pocket formed by the side chains of Phe170, Val173, and the main chain atoms of residues 166–168 (Figure 2C). These yield a stabilization of −7.5 kcal/mol to the complex.

A sulfate ion is tightly coordinated in the close vicinity of the inhibitor by direct hydrogen bonds contributed by the side chains of Asn292 and Asp307 and a water-mediated hydrogen bond with the main chain of Glu291 and the side chain of Asn244. This sulfate contributes with an oxygen−π interaction also involving the chlorophenyl group of CX-4945. The second sulfate ion is coordinated by Ser169 from the glycine-rich loop and Lys289 from the catalytic loop. The second ion is in a position similar to a hydrolyzed γ-phosphate from ATP bound to PKA (PDB 1RDQ)^46^ or a bound phosphate in the structure of Haspin with a 5-iodotubercidin ligand (PDB 3IQ7).^47^ It is plausible that targeting this conserved binding pocket containing positively charged amino acids by more druggable bioisosteres could improve the selective inhibitor design. Alternatively, the present sulfate ions could be exploited for the formation of additional polar interactions.

### Structural Basis of GSK3β Inhibition by CX-4945: Binding to the ATP Pocket

The binding of CX-4945 to both phosphorylated and non-phosphorylated (Tyr216) forms of GSK3β was characterized by protein crystallography. The kinase phosphorylated at the activation loop crystallized in the P3121 space group with two protein molecules in the asymmetric unit, while the non-phosphorylated kinase crystallized in the P43212 space group with a single protein molecule in the asymmetric unit. The structures were refined at 3.00 and 2.85 Å, respectively. Interestingly, both structures were determined from identically prepared kinase samples. Only the crystallization conditions allowed the separation of the phosphorylated from the non-phosphorylated form.

The overall structure of GSK3β adopts a classical bilobal kinase fold. The structures of the phosphorylated and non-phosphorylated forms of GSK3β superimpose with an RMSD of 0.41 Å over 252 C_α_ atoms, and in both cases, the ATP pocket represents the type I active kinase, DFG-in conformation. Irrespective of the structure, CX-4945 is located at the ATP-binding pocket (Figure S2I–L), and the binding mode is similar to that observed for the active site of the DYRK1A kinase (Figure 2E). The inhibitor is stabilized by three direct hydrogen bonds, water-mediated contacts, and hydrophobic interactions. The carboxyl moiety of the inhibitor contributes with a direct hydrogen-bond interaction with the amide of Asp200 (equivalent to Asp307 in DYRK1A) from the DFG motif (Figure 2E). This interaction mirrors quite closely the case of DYRK1A with the calculated ligand–residue binding energies between −13.1 and −14.7 kcal/mol depending on the selected molecular complex. In GSK3β, there is a hydrogen bond coupled with an ionic bridge to the side chain ammonium of Lys85 (Lys188 in DYRK1A, Figure 2E). Again, the interaction strength goes hand in hand with what was observed for DYRK1A, though in one of the protein–ligand complexes of GSK3β, the residue Lys85 is bridging between the ligand and Glu97. Such a bridging situation, in which two carboxylates compete for different hydrogen atoms of the same ammonium group, weakens the ligand–Lys85 contact by almost 4.0 kcal/mol. In the other complex of the asymmetric unit, the interaction between the ligand’s carboxylate, Glu97 (Glu203 in DYRK1A), and the main chain amide of Phe201 (Phe308 in DYRK1A) is mediated by a molecule of water. Using our in-pocket optimization algorithm on the ligand–residue complex to optimize the hydrogen-bond network indicates that the molecule of water is primarily mediating the interaction between Glu97 (donor) and Phe201 (acceptor).^48^ The strongest interactions involving CX-4945’s carboxylate are thus expected to involve Asp200 and Lys85, generating an anchoring motif in GSK3β similar to what we observed for DYRK1A.

One of the nitrogen atoms of the benzo-naphthyridine group (atom 8) establishes a hydrogen bond with the main chain amide of Val135 (Leu241 in DYRK1A). This interaction seems to be weaker in GSK3β than in DYRK1A: −7.8 kcal/mol instead of −9.6 kcal/mol. Such a gain of 1.8 kcal/mol is to some extent correlated with the increase of the donor–acceptor distance in the hydrogen bond, which results from the natural dynamics of the system. However, differences in the respective side chains (the removal of methylene from leucine-to-valine) contribute with a decrease of the interaction strength by 1.0 kcal/mol. Nevertheless, the weight of the hydrogen bond for this ligand–residue interaction remains identical in both protein complexes (55%). The adjacent main chain carboxyl oxygens (Asp133 and Val135; Glu239 and Leu241 in DYRK1A) establish π-stacking interactions, which, according to our calculations, should stabilize the structure with about −4.8 kcal/mol. We stress that our evaluation is based on the total ligand–residue interactions. Consequently, also in this case, the leucine-to-valine conversion in the pocket affects the total interaction energies. The hydrophobic interactions with the inhibitor mainly involve the following side chains of the kinase (corresponding residues of DYRK1A are indicated in parentheses): Ile62 (Ile165), Ala83 (Ala186), Val70 (Val173), Leu132 (Phe238) of the N-lobe and Leu188 (Leu294) of the C-lobe. Tyr134 participates in a ring-stacking interaction with the inhibitor, reminiscent of a hydrophobic interaction provided by the side chain of Met240 in DYRK1A. Cys199 contributes with a sulfur−π interaction instead of the hydrophobic interaction with the side chain of Val306 at an equivalent site of DYRK1A (Figure 2F). Overall, the sum of all hydrophobic side chain contributions is estimated to be approximately −15.7 kcal/mol. This is lower in GSK3β than in DYRK1A, which may be rationalized by the cysteine residue.

**Figure 3:**
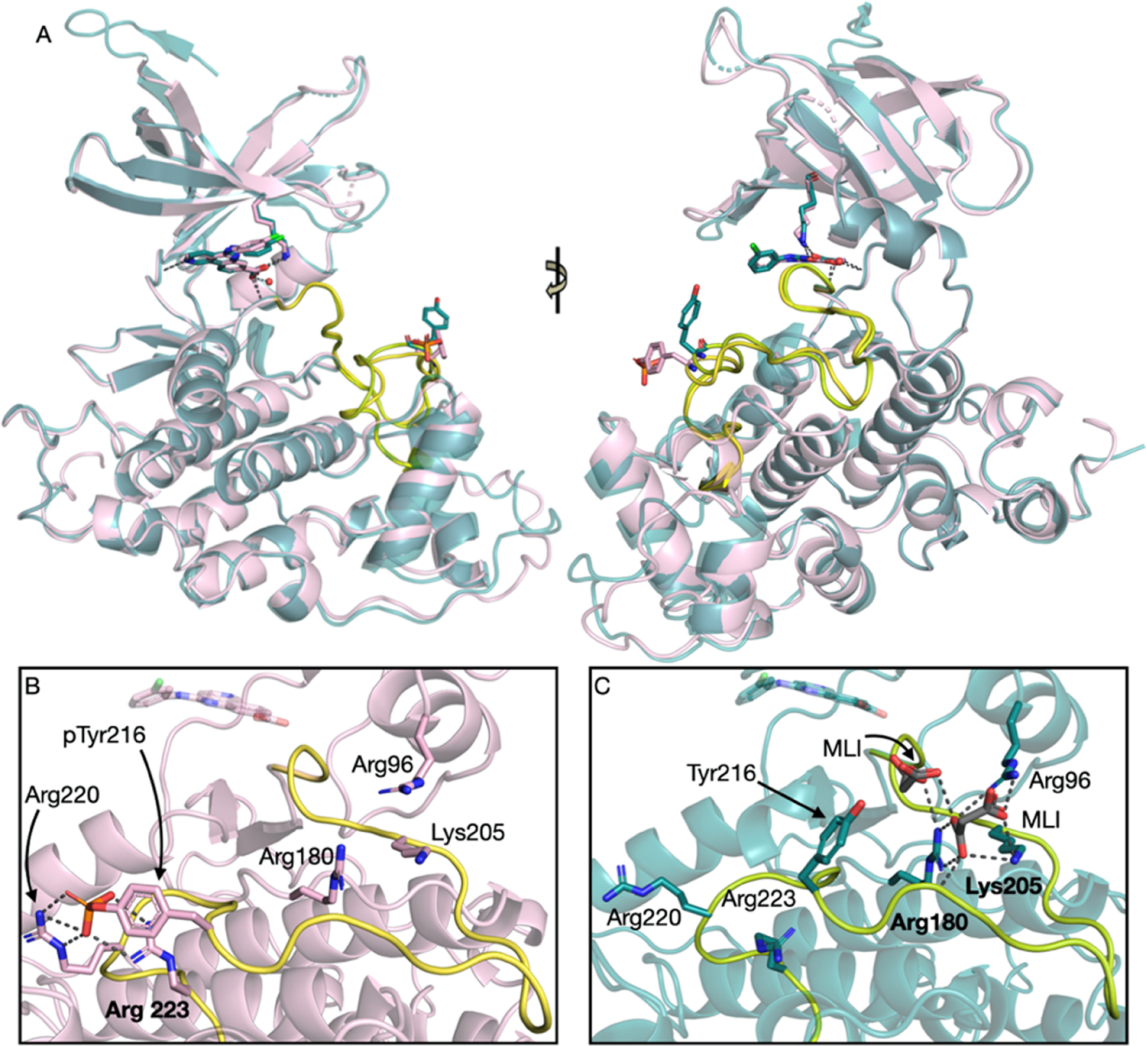
Activation loop comparison between phosphorylated and non-phosphorylated Tyr216 GSK3β forms. (A) Superimposition of phosphorylated (pTyr216, pink) and non-phosphorylated (Tyr216, teal) GSK3β with the CX-4945 inhibitor. The activation loop Asp200-Glu226 is shown in yellow. The stabilization of the activation loop in phosphorylated GSK3β (B) and non-phosphorylated GSK3β (C) form.

Comparing the crystal structures of Tyr216 phosphorylated and non-phosphorylated forms of GSK3β (Figure S2M,N) shows that major differences take place in the activation loop, more specifically, between residues 200 and 226 (Figure 3A). In the structures analyzed, the side chain of Tyr216 is seen in two distinct conformations. When Tyr216 is phosphorylated (pTyr216), the side chain of the residue is stabilized in an anti-conformation, which directs it out of the substrate binding site (Figure 3A). The phosphate moiety of pTyr216 makes interactions with Arg220 and Arg223, which helps in stabilizing the activation loop in the active conformation (Figure 3B). On the other hand, in the non-phosphorylated form of GSK3β, the side chain of Tyr216 is shifted toward the substrate binding groove between the two lobes of the kinase domain, thus adopting a gauche conformation. This directs the group downwards toward the bottom of the peptide binding cleft. The crystal structure for the non-phosphorylated form of GSK3β (crystalized in sodium acetate, imidazole, and disodium malonate) shows two malonate ions in the vicinity of Val214 (Figure 3C). These malonate ions form hydrogen bonds with three residues: Arg96, Arg180, and Lys205 from the substrate binding groove. The intense positive potential generated by the cluster of basic side chains is consequently neutralized. This neutralization of the positive charge and most significantly the interaction with Arg96 from the N-terminal lobe positions the catalytic residues in their active conformation. Calculations on model systems show furthermore that the presence of the malonates is critical for stabilizing the attachment of Tyr216 to the neighborhood of residues Arg96, Arg180, and Lys205. Once more, using in-pocket optimization to optimize the hydrogen-bond network, we observe that the malonates are bridging the tyrosine with the nearby arginine residues. Removing the malonates from the quantum chemical model system to better resemble the cell environment leads to an increase of the interaction energy by 1.9 kcal/mol. This means that the dissociation constant becomes more than 20 times higher. To better understand the phosphorylation of Tyr216, we used in-pocket optimization to generate a hypothetical structure of pTyr216 in the gauche conformation, thus interacting with Val214, Arg96, Arg180, and Lys205. Though, of course, our calculations do not incorporate the conformational changes accompanying the phosphorylation of gauche-fixed Tyr216, we observe a significant difference in the interaction with the neighboring residues in comparison to the anti-conformation. This suggests a strong thermodynamic driving force for flipping Tyr216 as soon as phosphorylation takes place.

In conclusion, both the phosphorylated and non-phosphorylated forms of GSK3β that we crystallized are in their active conformation. This is either supported by a hydrogen-bond network around pTyr216 or by exogenous oxyanions. The role of Tyr216 phosphorylation in GSK3β function has been uncertain, with contradictory results from *in vivo* studies.^49,50^ However, GSK3β kinase activity studies with phospho-primed peptide substrates revealed that Tyr216-phosphorylated GSK3β is only 5-fold more active than the corresponding non-phosphorylated enzyme.^51^ This is a very modest effect in comparison with related kinases, where activation segment tyrosine phosphorylation produces >1000-fold stimulation, suggesting that this particular phosphorylation has a modulatory, rather than a direct regulatory role in GSK3β function.

CX-4945 Restores DYRK1A/GSK3β-Mediated Inhibition of the Calcineurin/NFAT Pathway.

**Figure 4:**
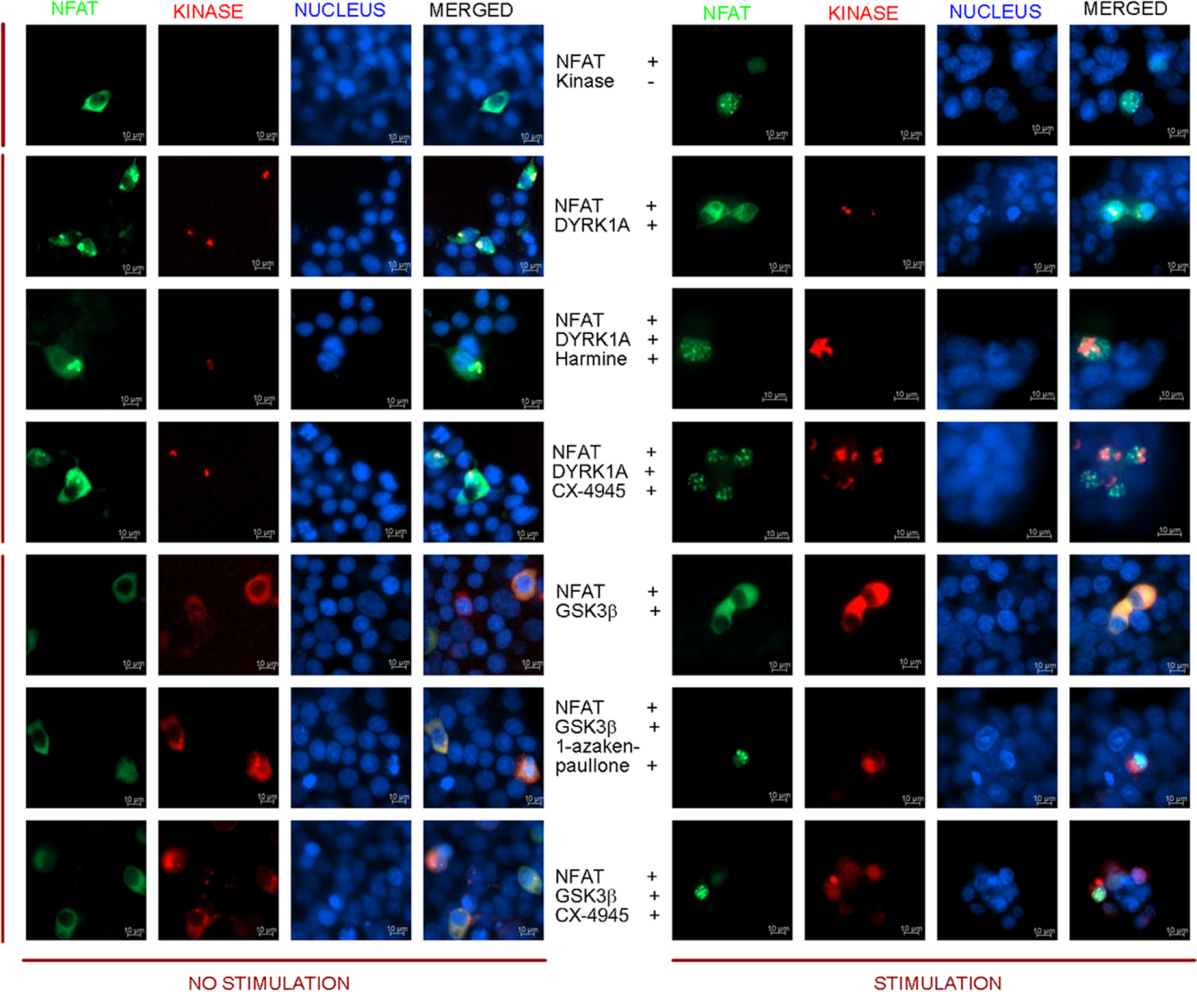
Representative fluorescence images of CX-4945’s impact on DYRK1A- and GSK3β-mediated inhibition on NFAT signaling. EGFP-NFATc1 (green) was cotransfected with either a mock vector, DYRK1A (red), or GSK3β (red) into HEK293T cells. Cells pretreated for 3 h with CX-4945 or harmine (5 μM) or DMSO before stimulation for 1 h with ionomycin (IM; 6 μM).

One of the important cellular targets of DYRK1A and GSK3β is the NFAT (Nuclear factor of activated T-cells) transcription factor, which plays a major role in regulating the cell cycle.^52^ The calcineurin/NFAT signaling controlled by DYRK1A and GSK3β activity is an important target for neurodegenerative processes and β-cell proliferation. Both kinases inactivate NFAT’s transcription factor by phosphorylation of its nuclear pool, which leads to cytosolic export. We therefore decided to check if treatment with CX-4945 could restore NFAT signaling via inhibition of DYRK1A or GSK3β. To assess the effect of CX-4945 on the calcineurin/NFAT/DYRK1A or GSK3β pathway, we imaged the nuclear translocation of the EGFP-NFAT fusion protein. In unstimulated cells, EGFP-NFAT stayed predominantly in the cytosol, and only after stimulation with ionomycin, the nuclear translocation and accumulation of EGFP-NFAT were observed (Figure 4). Overexpression of any of the kinases blocked the nuclear accumulation of EGFP-NFAT upon cell stimulation, proving the negative regulation of the calcineurin/NFAT pathway by DYRK1A or GSK3β. Treatment with either CX-4945 or harmine could restore the calcineurin/NFAT pathway and lead to the nuclear translocation and accumulation of EGFP-NFAT, despite the presence of overexpressed DYRK1A. Additionally, CX-4945 and 1-azakenpaullone reversed the effect of GSK3β-controlled NFAT cellular localization. These results further reinforce that CX-4945 efficiently inhibits DYRK1A and GSK3β in the cell and can restore the functionality of kinase-affected pathways.

### Kinase Selectivity Determinants of CX-4945

**Figure 5:**
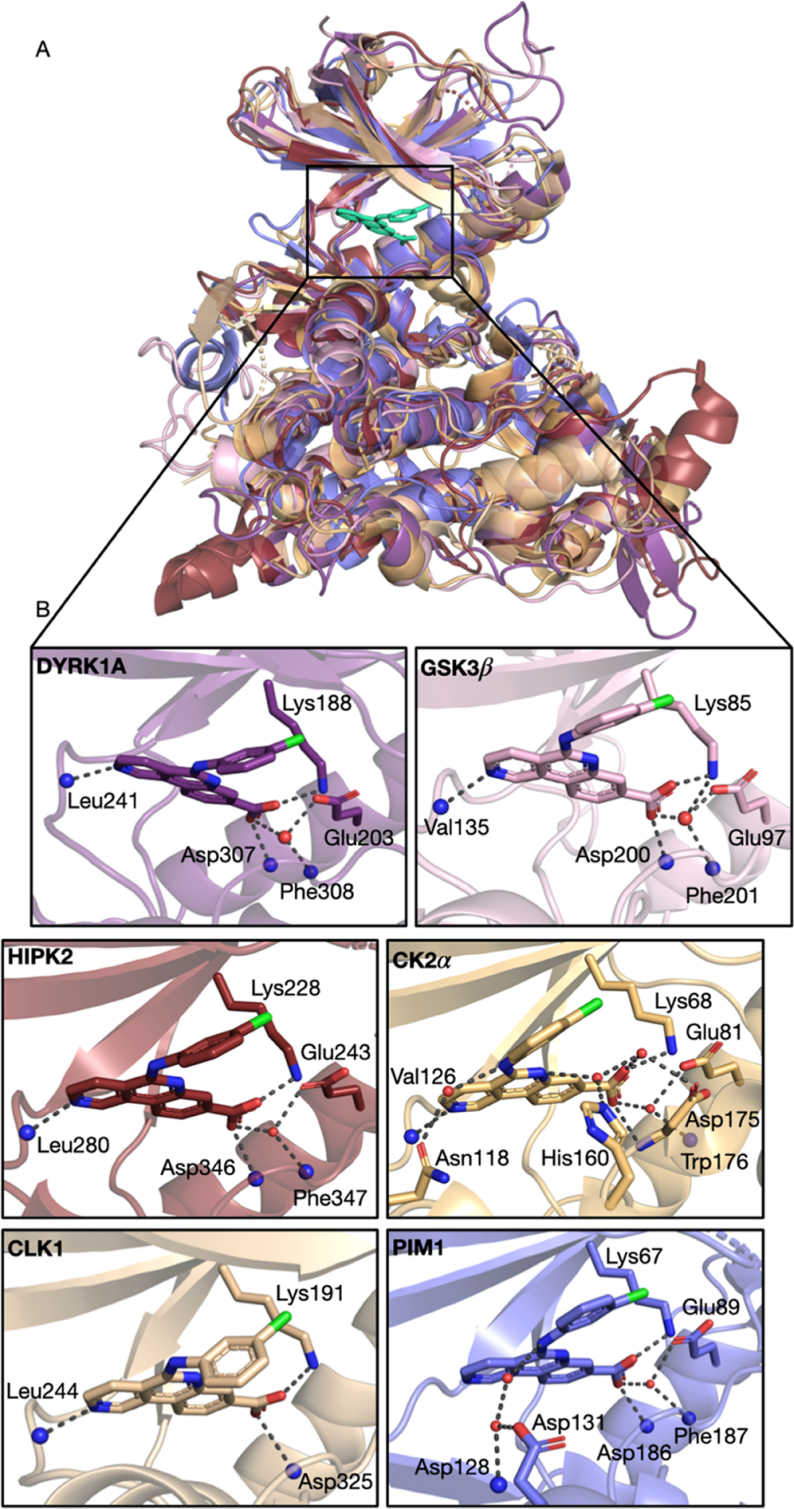
Binding mode of CX-4945 in ATP-binding pockets of CMGC and CAMK kinases. (A) Overlay of the crystal structures of kinases from CMGC and CAMK families crystalized with CX-4945. CX-4945 bound to DYRK1A (PDB ID 7Z5N, purple), GSK3β (PDB ID 7Z1F, pink), HIPK2 (PDB ID 6P5S,^34^ ruby), CK2α (PDB ID 3PE1,^10^ orange), CLK1 (PDB ID 6KHD,^35^ wheat), and PIM1 (PDB ID 5O11,^53^ slate). (B) Closeup of the binding mode of CX-4945 to the indicated kinases.

CX-4945 was previously characterized with respect to its selectivity profile. At a concentration of 500 nM, it affected the activity of 49 from the 235 kinases tested by more than 50%.^10^ However, only 10 kinases were inhibited by more than 90%. Four of them, CK2α, CLK3, DYRK2, and HIPK3, belong to the CMGC kinase family. DYRK1A was not included in the kinase panel selected for a single-concentration kinase screen during the initial evaluation, but its close isoform, DYRK2, was shown to be inhibited by more than 95%. For GSK3β, a 55% drop in activity was observed at 500 nM.^10^ Our study clearly demonstrates that CX-4945 directly inhibits DYRK1A and GSK3β at comparable nanomolar concentrations. Interestingly, the inhibitory activity of CX-4945 against DYRK1A was even stronger than that of harmine, an alkaloid obtained from plants and widely used as a selective and potent inhibitor of DYRK1A. The potent inhibitory activity of CX-4945 against CLKs,^35^ HIPKs, PIM1, and our two kinases, DYRK1A and GSK3β, is supported by structural data that provided detailed information on the binding mode of the inhibitor (Figure 5 and references therein). In all kinase inhibitor crystal structures, CX-4945 is firmly positioned in an ATP-binding pocket. Of the kinases with available crystal structures complexed with CX-4945, only PIM1 does not belong to the CMGC family but to CAMK. The different kinase family memberships are reflected in the binding mode of the inhibitor. In the CMGC family, CX-4945 forms three direct hydrogen bonds with the kinase protein, which involves a catalytic Lys, the amino group of Asp within the DFG motif, and the amino group of Leu or Val within the hinge region. In the PIM1 structure, no binding to the hinge region is observed because, contrary to the CMGC family members, PIM1 contains a Pro insertion in the hinge region. This reduces connections between the inhibitor and the kinase backbone. The anchoring of CX-4945’s carboxylate by a Lys residue is however conserved. Despite the similar binding poses of CX-4945 in these kinases, in particular, the ones in the CMGC family, protein–ligand complexes show different affinities.

**Figure 6:**
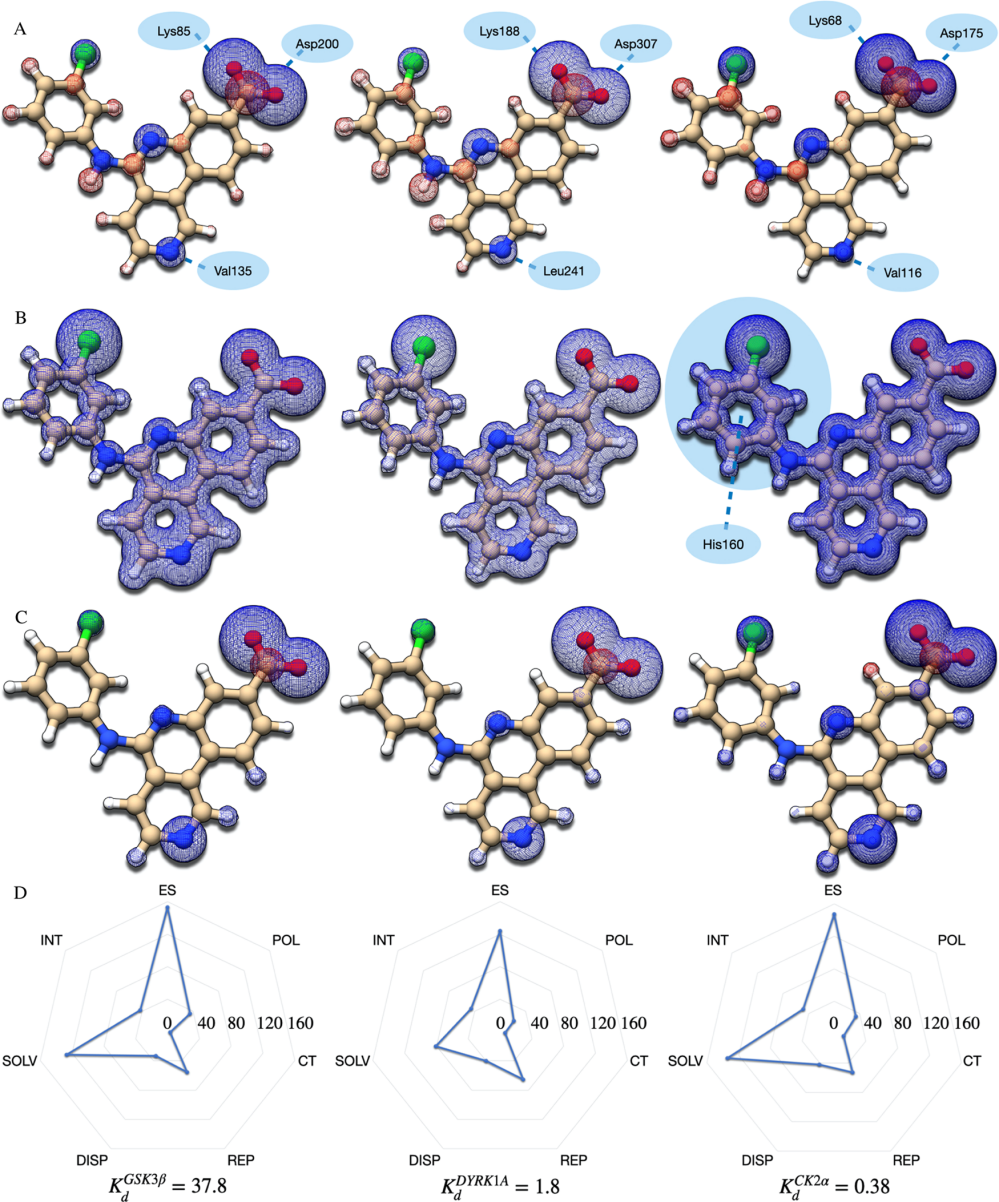
Energy decomposition analysis of the complexes between CX-4945 and GSK3β and DYRK1A and CK2α. (A) Maps for electrostatics; (B) lipophilicity maps based on dispersion interactions; (C) total interaction maps; and (D) the magnitude of different contributions to the energy decomposition. For each subfigure, the leftmost part relates to GSK3β, and in the middle, we have DYRK1A and then CK2α. Modeling was based on the PDB files 7Z1G (GSK3β), 7Z5N (DYRK1A), and 3PE1 (CK2α).

To better understand the origin of such differences, we applied our newly developed semiempirical energy decomposition analysis (EDA),^54^ which is suitable for in-depth analysis of binding energies. This was used to examine the binding modes of CX-4945 to DYRK1A and GSK3β and compare them to the original target of the inhibitor, CK2α. For better understanding, different contributions to the interaction energy are represented in the form of maps (Figure 6). These include electrostatics, dispersion forces (lipophilicity), and all summed contributions available in our EDA. Additional maps describing implicit solvation effects, exchange-polarization, overlap-repulsion, and charge transfer are presented in Figure S4. Values for each contribution to the binding energy are available in Table S5.

The first remark we make is that all interaction maps are very similar. This reflects the subtle differences expected for kinase selectivity.^55^ Electrostatic maps are strongly dominated by the ligand’s carboxylate group. This is due to the very strong hydrogen-bond interactions. There are additionally minor attractive contributions from aromatic nitrogen atoms. The remaining are untargeted long-range electrostatics. From the many calculations we performed on these three protein–ligand complexes, we verified that the partial charges on the carboxylate group are always well preserved: +0.35 electrons for the carbon and −0.60/–0.66 electrons for the oxygen atoms. Similar values were obtained for HIPK2, CLK1, and PIM1 and for the free ligand. This shows that despite its clear importance for binding, the different affinities all of these kinases show toward CX-4945 cannot possibly be accounted for by the carboxylate group and the respective hydrogen bonds. Finally, the magnitude of electrostatics allows us to infer that GSK3β offers the strongest (global) electrostatic effect over the ligand, followed closely by CK2α. DYRK1A offers a much weaker electrostatic stabilization of the ligand in the pocket. This further attests to our observation from above that electrostatics do not correlate with different binding affinities observed experimentally.

Interestingly, the dispersion maps for DYRK1A and GSK3β are indistinguishable (Figure S7 for a direct comparison), though the total lipophilic stabilization differs by almost 7 kcal/mol between proteins (DYRK1A is favored). Dispersion maps do however show quite clearly the delineation of the proteins’ shallow pockets as such close contacts result in stronger lipophilic interactions. In the case of DYRK1A and GSK3β, the benzo-naphthyridine ring is clearly the center of lipophilicity (*cf.*Table S6), though the strongest interactions per atom are on the chlorophenyl group (chlorine). Interactions with the benzo-naphthyridine are primarily dominated by ring C, in particular the atoms around nitrogen atom 8 (Ile165, Leu241, and Leu294 for DYRK1A; Ala83, Tyr134, and Leu188 for GSK3β). This is followed by ring A (Phe238, Val222, and Val306 in the case of DYRK1A; Cys199, Val110, and Leu132 for GSK3β), and finally by the amino-pyridine part, *i.e.*, ring B. The total contribution of dispersion is also revealing since it follows the trend of experimental affinities. Comparing total dispersion energies against the respective stabilizing effect of each functional group (Tables S6 and S7) reveals that, except for the phenyl group, CK2α and DYRK1A show quite similar behaviors. As London dispersion forces correlate with lipophilicity, we expect to a good extent for CK2α and DYRK1A to show identical lipophilic power. The CK2α and DYRK1A maps differ however in the relative contribution of the chlorophenyl group to the protein–ligand interactions. This is indicative of a very specific interaction that occurs in CK2α and is absent in DYRK1A. We then expect in CK2α an interaction targeting (or targeted by) the chlorophenyl moiety of the ligand. Comparatively, the global lipophilic character of GSK3β is significantly decreased. This is clear in the systematically lower interaction energies between each group and the protein, which is furthermore translated in the significantly lower total dispersion stabilization.

Consequently, we conclude that GSK3β offers a weaker lipophilic environment than the other two proteins. We stress furthermore that, consistent with all of these observations, there is a steep jump in the lipophilic contribution per atom in the ligand’s phenyl group when going from GSK3β to DYRK1A and then CK2α (Table S7). The information we extract from the dispersion maps extends however beyond the interactions between the π system of the inhibitor and the protein: it allows us to distinguish the different behavior evidenced by the carboxylate group. Despite its negative formal charge, carboxyls show reasonably soft oxygen atoms due to the delocalized double bond. It is therefore to expect that, contrary to alcohol, the carboxylate is also sensitive to the lipophilic environment of the protein. Though differences are not as astonishing as in the case of the previously discussed phenyl group, the carboxylate group of CX-4945 demonstrates stronger lipophilic affinity to DYRK1A and CK2α than it does for GSK3β. It is very intriguing to note that DYRK1A stabilizes this carboxylate even better by lipophilic interactions than CK2α. This hints at stronger lipophilicity of the protein in the part of the pocket associated with the carboxylate and is in good agreement with the potential targeted interaction between CK2α and the chlorophenyl group.

When summed up, the protein–ligand interactions are clearly dominated by the hydrogen bonds, which are identical in all complexes studied (*cf.*Figure 6C). This is in great agreement with thermodynamic binding data for CK2α bound to CX-4945,^56^ and it strengthens the anchoring picture built for the Asp and Lys residues conserved in all structures.^45^ In conclusion, the total interaction maps reinforce the critical role played by the carboxylate group and the nitrogen atom opposite to it in CX-4945’s scaffold (atom 8). Accounting for the different binding affinities requires however looking further into the strength of each interaction. Overall, our energy decompositions reveal that GSK3β and CK2α offer stronger electrostatic environments than DYRK1A. This is visible in Figure 6D and may furthermore be followed in Table S5. Figure S5 stresses the predominance of positively charged (blue) residues in the area around the inhibitor and provides a structural justification for the results of our calculations. Long-range electrostatics however lack the specificity to account for kinase selectivity. Our calculations support that the main forces that distinguish the binding of CX-4945 to GSK3β from binding to CK2α or DYRK1A are the dispersion interactions and the lipophilic environments of the proteins. This however does not account for the 5-fold difference in binding between CK2α and DYRK1A observed in our data.

**Figure 7:**
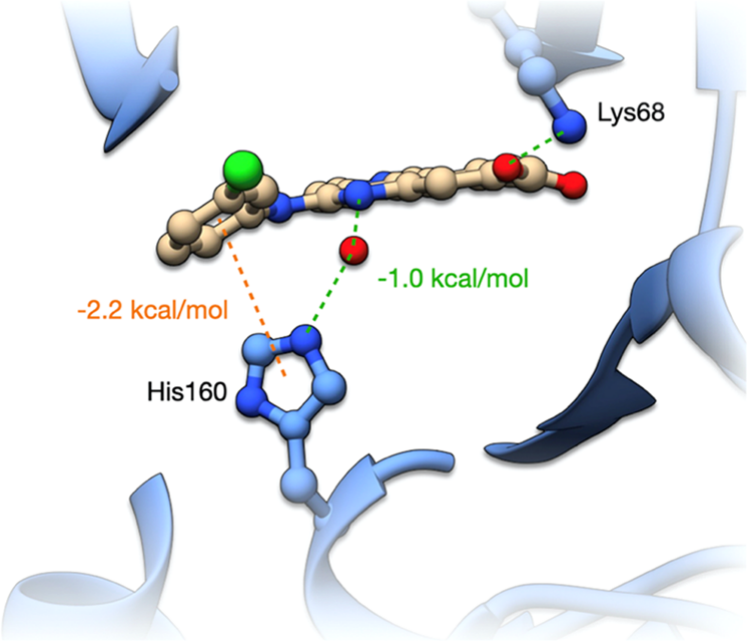
Interactions between His160 and CX-4945 in CK2α, as available from the PDB file 3PE1. Protein carbon atoms are represented in light blue, whereas the inhibitor’s carbon atoms are colored in light brown. Hydrogen bonds are marked in green dashed lines. We selected the anchoring of CX-4945’s carboxylate to Lys68 and the water-mediated hydrogen bond with His160. The orange dashed line marks the T-stack interaction.

Exploiting the short-range nature of London dispersion forces (*R*_AB_^–6^), we searched for the protein fragments with the strongest dispersion-like interactions with the chlorophenyl group of the inhibitor. Calculations on the first layer of amino acids of the pocket revealed that π-stacking with the main chain is an important factor for all proteins (for residues, see Table S8). In the case of CK2α, there is also a quite prominent T-stack contact with His160 (Figure 7) and the interaction with Leu45. Though the lipophilic contact with the alkyl chain of Leu45 is stronger in CK2α (than the contacts with the Ile residues in GSK3β and DYRK1A), these interactions are not specific to CK2α. Furthermore, these lipophilic contacts are rather unspecific and not directional. On the other hand, the T-stacked interaction between His160 and the ortho-chlorophenyl is exclusive to CK2α (*i.e.*, this interaction is absent in DYRK1A, GSK3β, HIPK2, all 4 CLKs, and PIM1) and it is quite direction-specific. We note that Battistutta and colleagues already detected the unusual orientation of His160 in this protein–ligand complex,^10^ but they assigned the effect to a water-mediated hydrogen bond between His160 and nitrogen 13 of ring B. Our calculations evidenced the role played by a T-stack interaction between His160 and the chlorophenyl moiety of the ligand. This inspired us to run a series of calculations on model systems built directly from the CK2α-CX-4945 crystal structure to infer which is the dominating contribution to the interaction between the two groups. All of the calculations resulted in a modest stabilization of approximately −1 kcal/mol for the water-mediated hydrogen bond between the imidazole ring and nitrogen 13. This is less than the stabilization achieved by the orientation-specific T-stack between the two aromatic rings (−2.2 kcal/mol). Furthermore, energy decomposition analysis of the complex with CK2α, including the crystal’s explicit waters, retains the exact same shape and weights observed for the interaction maps without explicit water molecules. This is particularly relevant to ascertain the weight attributable to the stabilizing effect of the water-mediated interaction with His160. Nevertheless, all structures of CK2α complexed with the CX-4945 position of the imidazole ring of His160 in a way that promotes the hydrogen-bond interaction.^10,56,57^ This is experimental evidence that the interaction is of importance to the unusual orientation of the side chain of His160. To ascertain the robustness of our calculations, we ran high-level *ab initio* calculations on the two complexes. Those calculations evidenced that the relative strength of the two interactions is dependent on the medium surrounding the protein–ligand complex. Cautiously, we state that in water, the two interactions are equally strong and that we expect the T-stack to dominate in more polar media. Further tests run on the same systems show furthermore that the T-stack interaction is very close to the optimal interaction mode between the two groups. More details on these two aspects may be found in the Supporting Material. The theoretical data that we collected points to the fact that the picomolar affinity of CX-4945 to CK2α is largely due to His160. The latter may thus be seen as an additional anchoring point of the ligand to the protein.

## Discussion

Small molecule kinase inhibitors are one of the most pursued goals in drug discovery. CX-4945, known as silmitasertib, was developed by Cylene Pharmaceuticals in 2011 and is one of the most promising drug candidates of this class. It also recently entered phase I/II clinical trials for cholangiocarcinoma^58^ and multiple myeloma.^6^ The therapeutic potential of CX-4945 arises due to its well-documented *in vitro* and *in vivo* efficiency and is also supported by its desirable pharmacokinetic profile (long half-life, oral bioavailability, limited toxicity).^59,60^ Here, we show that CX-4945 strongly binds and inhibits DYRK1A and GSK3β. Consequently, both kinases could be considered exploitable therapeutic targets. Since DYRK1A and GSK3β are a pair of priming and processive kinases, which sequentially phosphorylate common substrates, it is difficult to distinguish which kinase is the inhibitor target within the cell. For the assays, we selected Cyclin D1, which is a unique substrate of both kinases but not linked by priming activity. For both kinases, treatment with CX-4945 inhibits Cyclin D1 (Thr286) phosphorylation. Additionally, in the cells treated with CX-4945, we observed the recovery of DYRK1A- and GSK3β-controlled NFAT signaling. This proved that CX-4945’s inhibitory activity against the tested kinases is not limited to biochemical assays, and also CX-4945 is able to impair DYRK1A- and GSK3β-mediated phosphorylation in mammalian cells. The structural evaluation revealed subtle but deciding factors that determine the inhibitor specificity. However, it was possible only with the support of quantum chemical calculations to perform an in-depth analysis that pointed us toward kinase selectivity elements. We believe that this approach can be used broadly to predict and tune kinase inhibitor selectivity. Our calculations indicate that the different affinities of CX-4945 toward GSK3β and DYRK1A are related to the environment offered by each protein. In GSK3β, electrostatics dominate, a reflection not only of the nature of residues in the kinase’s pocket but especially of the overall charge distribution/environment provided by the whole protein. In DYRK1A, lipophilicity is stronger. Our calculations show that this is not a consequence of any residue in particular but rather a feature of the whole pocket. Further strengthening this observation is the increased shape complementarity offered by DYRK1A’s pocket, reflected in the increased REP contribution. This is essential for the strength of lipophilic contacts between the protein and the inhibitor. The calculations show that CX-4945 favors binding to lipophilic environments, which accounts for the relative affinity exhibited toward GSK3β, DYRK1A, and CK2α. This provides further support to observations made by others^10^ that lipophilicity is a key feature in targeting the ATP-binding pocket of CK2α. Besides two anchoring hydrogen bonds between the ligand and the protein, no other interaction came out as particularly strong from our analysis. Our calculations suggest that if kinase-specific inhibitors are to be developed, then investing in the electrostatic prowess of the inhibitor will be favorable to strengthen binding to GSK3β. On the other hand, further endowing the ligand with lipophilicity is expected to promote binding to DYRK1A. However, it is important that the two key hydrogen-bond interactions remain unaffected. To account for the subnanomolar affinity of CX-4945 to CK2α, we invoke a targeted interaction with a histidine residue exclusive to this protein. Our calculations suggest that the T-stacked interaction of this residue with the chlorophenyl fragment of the inhibitor is quasi-optimal and dominates over water-mediated hydrogen bonds in polar media. Nonetheless, we feel that both interactions are important to create an additional anchor of the ligand to the protein. Also, according to the calculations, further development of the inhibitor should focus on this contact. The interaction maps show that investing in the lipophilic character of the chlorophenyl group should promote the affinity of the ligand to CK2α. The quantum chemical calculations on model systems reveal that the His-chlorophenyl T-stacked interaction is optimal when the H_ε1_ proton of His is positioned in the center of chlorophenyl’s aromatic ring. Though we did not include entropic effects in these calculations, a rough estimate indicates that modifications of CX-4945 that achieve that structural arrangement could improve binding by a factor of 3.

Overall, our results further suggest that CX-4945 may be considered as an interesting, proof-of-concept molecule to study both *in vitro* and *in vivo* regulation of metabolic pathways dependent on DYRK1A and GSK3β kinase activity. For example, related to diabetes or neurodegenerative diseases. The confirmed safety, high affinity, and reasonable selectivity make this a viable candidate for development up to clinical trials.

In addition, future modifications of the CX-4945 scaffold are desirable to achieve higher efficacy and selectivity. We are convinced that our structural and quantum mechanical data will serve as a solid basis for further medicinal chemistry development.

## Conclusions

We investigated the affinity of a clinical casein kinase 2 inhibitor, CX-4945, toward the DYRK1A and GSK3β kinases implicated in the biology of several diseases. The results confirmed that CX-4945 strongly binds to DYRK1A and GSK3β kinases with a dissociation constant (*K*_d_) of 1.8 and 37.8 nM while being a very potent inhibitor of both kinases with IC_50_ in the nanomolar range (160 and 190 nM). Inhibitory activity toward both kinases was not only limited to *in vitro* assays but also extended to cellular models. The activity of this inhibitor toward other kinases offered the opportunity to study the effect of DYRK1A and GSK3β kinases on the NFAT pathway, allowing us to predict a positive effect toward β-cell expression and diabetes. The crystal structures of CX-4945 complexed with DYRK1A and GSK3β were solved by X-ray crystallography and analyzed using additional quantum chemical models. Extending our analysis to CK2α, we built a quantum chemical selectivity model using our new energy decomposition analysis. Only with the help of extensive quantum chemical calculations could we identify a key element for CK2α’s subnanomolar affinity to CX-4945, which may be exploited in future drug discovery ventures. This is a particular contact between the chlorophenyl group of CX-4945 and His160 of the protein (Figure 7). The methodology we employed is expandable to other kinase selectivity modeling.

## Experimental Section

### Compound Purity

All compounds are >95% pure by HPLC. Harmine, acquired from Sigma-Aldrich, catalog number 286044, purity of 98%; 1-azakenpaullone, acquired from Sigma-Aldrich, catalog number A3734, purity of 96.5%; CX-4945, acquired from MedChemExpress, catalog number HY-50855, purity of 99.3%.

### Cell Culture

The human HEK293T cell line was obtained from the European Collection of Cell Culture. Cells were cultured in minimal DMEM medium (Invitrogen) supplemented with 10% fetal bovine serum (Lonza). Cells were maintained at 37 °C in a humidified atmosphere containing 5% CO_2_.

### Plasmid Construction

The DNA encoding kinase domains of both DYRK1A (126–490) and GSK3β (26–383) with N-FLAG (MDYKDDDDK) and NFATc1 and Cyclin D1 with N-HA (MYPYDVPDYS) tags were synthesized by Genscript and cloned into pcDNA3.1 for eukaryotic cell overexpression. Fluorescent protein fusions were prepared by appending genes encoding mCherry and EGFP using restriction-free cloning.^61,62^

For bacterial expression, the fragment of the gene encoding kinase domain of DYRK1A (126–490) was PCR amplified and subcloned into pET24a, using a restriction-free method.^61,62^ The kinase domain of DYRK1A was expressed together with a non-cleavable C-terminal hexahistidine tag. The fragment of the gene encoding kinase domain of GSK3β (26–383) was codon optimized and synthesized by Genscript, and then the gene was cloned into a pET24a expression plasmid. The kinase domain of GSK3β was expressed with a C-terminal hexahistidine tag and proceeded with tobacco etch virus protease cleavage site (ENLYFQ*GHHHHHH).

### Protein Expression and Purification

DYRK1A was expressed in E. coli LOBSTR strain (Kerafast) in LB medium supplemented with kanamycin (50 μg/mL) at 17 °C for 16 h. The pellet was resuspended in cold lysis buffer (20 mM HEPES pH 7.5, 500 mM NaCl, 5% glycerol, 15 mM imidazole, and 5 mM 2-mercaptoethanol supplemented with EDTA-free Protease Inhibitor Cocktail (Roche)) and the cells were disintegrated by sonication. Clarified lysate was passed through HisPur Cobalt resin (Thermo Fisher Scientific, Waltham, MA, United States), and the protein of interest was eluted with stepwise increments of imidazole concentration (50–300 mM). The fraction corresponding to DYRK1A was pulled and dialyzed against 20 mM HEPES, pH 7.5, containing 50 mM NaCl and 5 mM 2-Mercaptoethanol. Further purification was obtained by ion-exchange chromatography on a HiTrap Q FF column (Cytiva) followed by size exclusion chromatography on a HiLoad 16/600 Superdex 75 pg column (Cytiva) in 20 mM HEPES, pH 7.5, containing 150 mM NaCl and 5 mM 2-mercaptoethanol. Purified DYRK1A kinase was flash-frozen in liquid nitrogen and stored at −80 °C for further analysis.

The initial expression and purification steps for GSK3β were identical to DYRK1A expression, except that the TB medium was used instead of LB, and the buffers used were of pH 7.2 instead of 7.5. The His tag was removed by TEV protease cleavage during dialysis, subsequent to affinity resin elution. The separated His tag was removed by negative chromatography on HisPur Cobalt resin. Further purification and buffer exchange was obtained on a HiLoad 16/600 Superdex 75 pg column (Cytiva). Purified GSK3β was flash-frozen in liquid nitrogen and stored at −80 °C for further analysis.

For the His-tagged GSK3β, the purification was similar to DYRK1A, except that the buffers used were of pH 7.2 instead of 7.5, and the final buffer for size exclusion chromatography contained 300 mM NaCl instead of 150 mM. Purified His-tagged GSK3β was flash-frozen in liquid nitrogen and stored at −80 °C for further analysis.

### Microscale Thermophoresis and Ratiometric Analysis

Human kinases (GSK3β and DYRK1A) were labeled using Monolith His-Tag Labeling Kit RED-tris-NTA 2nd Generation (MO-L018, NanoTemper Technologies) according to the manufacturer’s instructions, and labeled proteins are referred to as targets. The affinity of the dye to the his-tagged proteins was estimated prior to the binding assay according to the manufacturer’s instructions. Minimal target concentrations in the assay were set to a constant concentration of 20 nM for DYRK1A and 62.5 nM for GSK3β-based on the evaluation of the dye-target interaction analysis. In the binding assays, small molecules were used as ligands in three parallel two-fold dilution series, with starting concentrations 5, 5, and 100 μM in the case of harmine, CX-4945, and 1-azakenpaullone for DYRK1A, respectively, and 200, 12.5, and 50 μM in the case of harmine, CX-4945, and 1-azakenpaullone for GSK3β, respectively. Microscale thermophoresis and ratiometric measurements were performed simultaneously on a Monolith X (NanoTemper Technologies), at wavelengths 670 and 650 nm, with medium MST power.^63^ Dissociation constants (*K*_d_) were analyzed using the MO Control software (NanoTemper Technologies). Data were visualized in the OriginPro 2022 software package.^64^

### Cook Activity Assay

The inhibitory potency (IC_50_) of all compounds was determined in the Cook activity assay^39,62^ in which ADP production was coupled to NADH oxidation by pyruvate kinase and lactate dehydrogenase. The peptide substrates, DYRKtide (RRRFRPASPLRGPPK) for DYRK1A and GYS1 (YRRAAVPPSPSLSRHSSPHQ(pS)EDEEE) for GSK3β, were chemically synthesized by Caslo ApS. The assay mixture contained 100 mM MOPS (pH 6.8), 100 mM KCl, 10 mM MgCl_2_, 1 mM phosphoenolpyruvate, 1 mM peptide substrate, 1 mM 2-mercaptoethanol, 15 U/mL lactate dehydrogenase with 10 U/mL pyruvate kinase, and 10.7 mM NADH. Seventy-five microliters of the assay mixture was mixed with 10 μL of 2.5 μM kinase and 5 μL of a compound in DMSO with a concentration ranging from 20 nM to 200 μM and incubated for 10 minutes at room temperature. Then, the reaction was started by the simultaneous addition of 10 μL of 1280 μM ATP. The enzyme velocity was measured at 340 nm over a time period of 300 s at room temperature. Control reactions in the absence of the peptide substrate were used to detect ATPase activity for basal concentrations. All measurements were done in triplicate, and IC_50_ was determined using GraphPad Prism software.

### Dye-Based Thermal-Shift Assay

DYRK1A and GSK3β stability in the presence of harmine, CX-4945, and 1-azakenpaullone were analyzed by the proteins’ melting temperature determination using the thermal-shift assay (TSA) as described previously.^40^ Both proteins (1.5 mg/mL) were incubated with a 1:200 diluted Sypro Orange dye, 20 mM HEPES, 100 mM KCl, 10 mM MgCl_2_, 1 mM 2-mercaptoethanol (pH 8.0) and compound (10 μM) or DMSO. The fluorescence signal of Sypro Orange was determined as a function of temperature between 5 and 95 °C in increments of 0.5 °C/min (λ_ex_ 492 nm, λ_em_ 610 nm). The melting temperature was calculated as the inflection point of the fluorescence as a function of temperature. Each experiment was carried out in triplicates.

### Protein Crystallization, Data Collection, and Structure Determination

For crystallization, DYRK1A was concentrated to 12–15 mg/mL and GSK3β to 6–8 mg/mL. The proteins were incubated overnight with 3–10 molar excess of CX-4945 at 4 °C. The preparation was mixed 1:1 (v/v) with the crystallization solutions. Crystallization experiments were carried out at 20 °C. Crystals appeared within 1–3 days at room temperature.

The DYRK1A/CX-4945 complex (PDB ID 7Z5N) was obtained in 0.1 M Tris HCl (pH 7.7) containing 0.1 M lithium sulfate and 40% PEG400.

The phosphorylated GSK3β/CX-4945 complex (PDB ID 7Z1F) was obtained in 0.1 M imidazole (pH 6.5) containing 1.0 M sodium acetate trihydrate and 0.2 M and 10 mM yttrium (III) chloride.

The non-phosphorylated (Tyr216) GSK3β/CX-4945 complex (PDB ID 7Z1G) was obtained in 0.1 M imidazole (pH 6.5) containing 1.0 M sodium acetate trihydrate and 0.2 M disodium malonate.

Crystals were cryoprotected with mother liquor containing 25% glycerol and flash-frozen in liquid nitrogen. The diffraction data were collected at BESSY (Berlin) and ESRF (Grenoble).

The diffraction data was indexed and integrated in XDS.^65^ Data was scaled in AIMLESS^66^ from the CCP4 software package.^67^ Following steps were performed in Phenix.^68^ The structures of DYRK1A and both GSK3β were solved by molecular replacement using PHASER^69^ and 6EIS and 6GN1, respectively, as search models. Models were refined by interchanging cycles of automated refinement using phenix.refine^70^ and manual building in Coot.^71^ Data collection and refinement statistics are summarized in Table S2. Restraints for the inhibitors were created in the GradeServer.^72^

### Cyclin D1 Phosphorylation Profile

HEK293T cells were seeded in 24-well plates at the density of 2×105 cells/well. Twenty-four hours later, the cells were cotransfected with plasmids encoding HA-Cyclin D1 (0.5 μg) with Flag-DYRK1A or FLAG-GSK3β or an empty vector (0.2 μg) using PEI Prime (Sigma-Aldrich). Forty-eight hours later, the cells were treated with inhibitors CX-4945 (10 μM), harmine (10 μM), 1-azakenpaullone (10 μM), or DMSO for 3 h and lysed in RIPA buffer containing protease inhibitors (Sigma-Aldrich) and phosphatase inhibitors (Calbiochem). Total cell proteins (10 μg) were separated by 12% SDS-PAGE and transferred to the PVDF membrane (ThermoScientific). Proteins were analyzed by Western Blot using the anti-FLAG monoclonal antibody (Sigma-Aldrich; F3165) for DYRK1A and GSK3β detection, the anti-HA monoclonal antibody (Cell Signaling; C29F4) for Cyclin D1, and anti-phospho-Cyclin D1 (Thr286) (Cell Signaling; D29B3) for phospho-Cyclin D1 relevant HRP-conjugated secondary antibodies.

### NFAT Translocation Assay

HEK293T cells were grown on a μ-Slide 8 well (IBIDI) to 50%–70% confluency. The plasmids expressing the desired proteins, mCherry-DYRK1A or mCherry-GSK3β and eGFP-NFATc1, were transiently cotransfected for 24 h with PEI Prime (Sigma-Aldrich). Cells were pretreated with inhibitors CX-4945 (5 μM), harmine (5 μM), or DMSO for 3 h and then stimulated with ionomycin (Thermo Fisher Scientific) for 1 h. Cells were washed with 1 mL of PBS, and the nuclei were stained with Hoechst 33258 (ThermoScientific) for 10 min at 37 °C and fixed with 4% paraformaldehyde in phosphate-buffered saline (PBS) for 10 min at 25 °C. Images were collected with a Zeiss Axio Observer 3 fluorescence microscope and analyzed in ZEN Blue edition software.

### Computational Studies

Most quantum chemical calculations were performed with the ULYSSES package.^73^ The method of choice was GFN2-xTB^74^ together with the ALPB solvation model,^75^ which we showed in a recent publication to be adequate to accurately account for nonbonded interactions in systems of biological interest.^54^ See also the Supporting Material for further discussion, taking also into consideration the recent work of Villot and co-workers^76^ on the suitability of GFN2-xTB when applied to biological systems. When mentioned, electronic populations were estimated using Mulliken population analysis. In the optimization of fragments of the experimental structures, we used our recently developed in-pocket optimization, and the energy decomposition analysis was performed according to our recent method.^48,54^ The proteins were prepared using Schrödinger’s MAESTRO,^77^ followed by OPLS3e structural minimization.^78^ The pockets were all cut manually to understand and ensure their balance with respect to the full protein systems.

DLPNO-CCSD(T)^79^ calculations were run with ORCA 5.0 with libint2 using the default options.^80^ Calculations were run using the def2-TZVP^81^ basis set with the resolution of identity.^82^

## Supporting Information Available

Output of our EDA calculations as well as the maps for each protein–ligand complex studied (ZIP)
Data associated with model system calculations (ZIP)
Collected physicochemical data on silmitasertib, crystallographic data, dye-based thermal-shift assay, quantum chemical calculations on model systems, additional interaction maps, the balance of quantum chemical calculations (PDF)

(ZIP)
(ZIP)
(PDF)

## Abbreviations Used

ADP: Adenosine diphosphate
ATP: Adenosine triphosphate
CAMK: Ca^2+^/calmodulin-dependent protein kinase
CK2: casein kinase 2
CLK1: cyclin-dependent kinase-kike kinase 1
CLK2: cyclin-dependent kinase-kike kinase 2
CLK3: cyclin-dependent kinase-like kinase 3
CLKs: cyclin-dependent kinase-like kinases
CMGC: CDK/MAPK/GSK/CDK-like kinase
DMSO: dimethyl sulfoxide
DNA: deoxyribonucleic acid
DYRK1A: dual-specificity tyrosine phosphorylation-regulated kinase 1A
EDA: energy decomposition analysis
EDTA: ethylenediaminetetraacetic acid
EGFP: enhanced green fluorescent protein
EPKs: eukaryotic protein kinases
FBS: fetal bovine serum
FDA: Food and Drug Administration
FLT3: FMS-like tyrosine kinase 3
GFP: green fluorescent protein
GSK3β: glycogen synthase kinase 3 β
HEK293T: human embryonic kidney cell line 293T
HEPES: *N*-2-hydroxyethylpiperazine-*N*′-2-ethanesulfonic acid
HIPK2: homeodomain-interacting protein kinase 2
HRP: horseradish peroxidase
IC_50_: half maximal inhibitory concentration
IM: ionomycin
*K*_d_: dissociation constant
*K*_i_: inhibition constant
*K*_m_: Michaelis-Menten constant
MST: microscale thermophoresis
NADH: nicotinamide adenine dinucleotide
NFAT: nuclear factor of activated T cells
NFATc1: nuclear factor of activated T-cells, cytoplasmic, calcineurin-dependent 1
PBS: phosphate-buffered saline
PBST: phosphate-buffered saline with Tween 20
PIM1: serine/threonine-protein kinase pim-1
PKA: protein kinase A
PMA: phorbol 12-myristate 13-acetate
SMKI: small molecule kinase inhibitors
