# Supplementary material for: Silmitasertib (CX-4945), a Clinically Used CK2-Kinase Inhibitor with Additional Effects on GSK3β and DYRK1A Kinases: A Structural Perspective
Source: J Med Chem. 2023 Mar 8;66(6):4009–24. doi: 10.1021/acs.jmedchem.2c01887 (PMC10041529; doi:10.1021/acs.jmedchem.2c01887)
Supplement: Supplementary file 1 — jm2c01887_si_001.zip [file jm2c01887_si_001.zip › EDA/README.docx]

For each folder, open the structure file (PDB) in pymol or chimera. Then open the maps, also with pymol or chimera. For the purpose of representation, in chimera we always used a resolution of 0.001.

The maps are the cub files. The maps with suffix "_minus" correspond to the attractive part of the interaction, i.e., the blue spheres in the figures of the paper. The ones with suffix “_plus” correspond to the repulsive part (red). For some interactions there are no maps. For instance, dispersion is always attractive, therefore the “DISP_plus” maps are empty. The same applies to “REP_minus” (repulsion is repulsive). We left these nonetheless, because it makes easier in chimera to colour the maps if opened in a certain order.

The *.out files contain the output of our EDA calclations. You have the calculation of the intermediate states and then the printing of the interaction matrices. This is followed by the report of the interaction energies.
