# Supplementary material for: Silmitasertib (CX-4945), a Clinically Used CK2-Kinase Inhibitor with Additional Effects on GSK3β and DYRK1A Kinases: A Structural Perspective
Source: J Med Chem. 2023 Mar 8;66(6):4009–24. doi: 10.1021/acs.jmedchem.2c01887 (PMC10041529; doi:10.1021/acs.jmedchem.2c01887)
Supplement: Supplementary file 3 — jm2c01887_si_003.pdf [file jm2c01887_si_003.pdf]

Supporting Information

Silmitasertib (CX-4945), a clinically used CK2-kinase inhibitor with additional effects on GSK3β and DYRK1A kinases – a structural perspective

Przemyslaw Grygier[1‡], Katarzyna Pustelny[1‡], Jakub Nowak[1], Przemyslaw Golik[2], Grzegorz M. Popowicz[3,4], Oliver Plettenburg[5,6,7,8], Grzegorz Dubin[1], Filipe Menezes[3,4,*], Anna Czarna[1,*]

[1]Malopolska Centre of Biotechnology, Jagiellonian University, Gronostajowa 7A, 30-387 Krakow, Poland

[2]Selvita S.A, Bobrzynskiego, 14, 30-338 Krakow, Poland

[3]Institute of Structural Biology, Helmholtz Zentrum Muenchen, Ingolstaedter Landstrasse 1, Neuherberg 85764, Germany

[4]Biomolecular NMR and Center for Integrated Protein Science Munich at Department Chemie, Technical University of Munich, Lichtenbergstrasse 4, Garching 85747, Germany

[5]Institute of Medicinal Chemistry, Helmholtz Munich, Ingolstaedter Landstrasse 1, Neuherberg 85764, Germany

[6]Institute of Organic Chemistry, Centre of Biomolecular Drug Research (BMWZ) and Laboratory of Nano and Quantum Engineering (LNQE), Leibniz University Hannover, Schneiderberg 1b, Hannover 30167, Germany

[7]German Center for Diabetes Research (DZD), Ingolstaedter Landstrasse 1, Neuherberg 85764, Germany

[8]Institute of Lung Health (ILH), Aulweg 130, Giessen 35392, Germany

‡ Contributed equally

*Correspondence: anna1.czarna@uj.edu.pl filipe.menezes@helmholtz-muenchen.de

Table of Contents

S1 – Silmitasertib's physico-chemical properties……………………………………………….S2
S2 – Crystallographic Data………………………………………………………………….S2
S3 – Dye-based Thermal Shift Assay………………………….……………………………...S5
S4 – Quantum Chemistry on Small Protein-Ligand Complexes……………………………….S5
S5 – Interaction Maps for CX-4945 in different proteins………………………………………S7
S6 – Overall Balance in Quantum Mechanical Calculations…………………………………...S12

SUPPLEMENTAL INFORMATION

S1 – Silmitasertib's physico-chemical properties

Table S1. Summary of physico-chemical properties of the inhibitor CX-4945.

Property	Value	Source
Molecular weight	349.77 g/mol	SwissADME
Water Solubility	2.34e-03 mg/ml	SwissADME
Log S (ESOL)	-5.17	SwissADME
Log Po/w (MLOGP)	3.28	SwissADME
Log D7.4	3.46	1
Hydrogen Acceptor Count	5	SwissADME
Hydrogen Donor Count	2	SwissADME
Polarizability (Å^3)	35.7	Calculated in ULYSSES
Polar Surface Area	75.11 Å2	2
LiPE (CK2α)	6.1	

S2 – Crystallographic Data

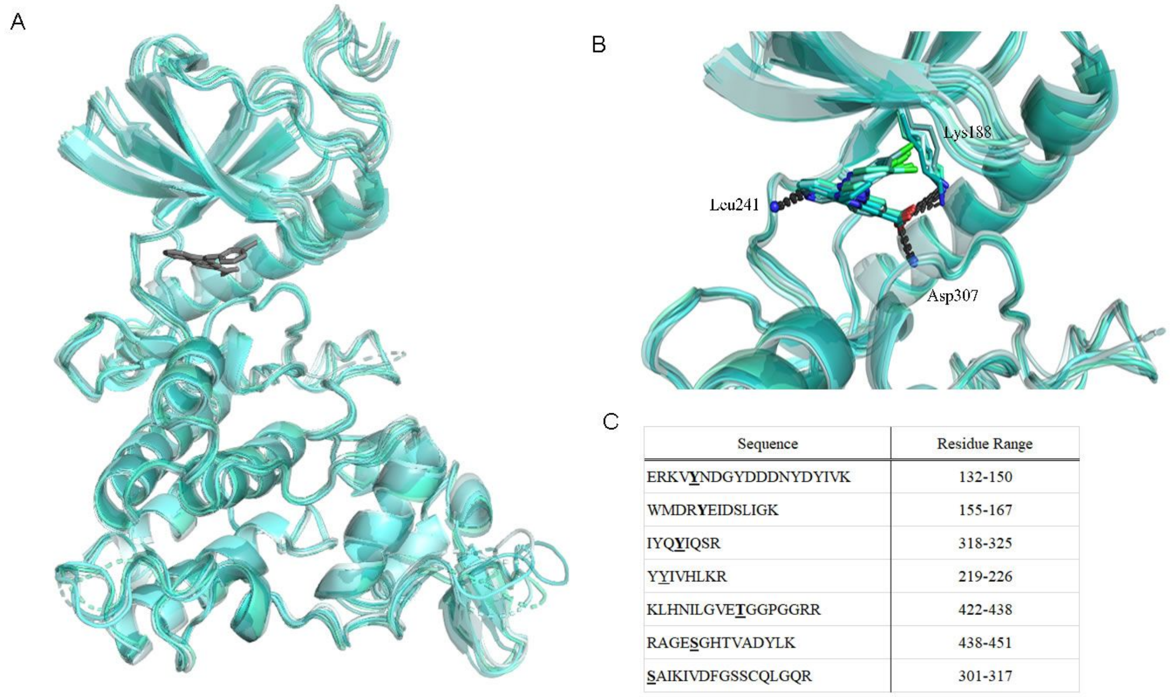

C

Sequence	Residue Range
ERKV**Y**NDGYDDDNYDYIVK	132-150
WMDR**Y**EIDSLIGK	155-167
IYQ**Y**IQSR	318-325
Y**Y**IVHLKR	219-226
KLHNILGVE**T**GGPGGRR	422-438
RAGE**S**GHTVADYLK	438-451
**S**AIKIVDFGSSCQLGQR	301-317

Figure S1. Structural comparison of DYRK1A molecules found in the asymmetric unit (A) Superimposition of eight DYRK1A molecules found in the asymmetric unit: 1, cyan; 2 palecyan; 3, aquamarine; 4, greencyan; 5, teal; 6, paleteal; 7 lightteal; 8, deepteal. (B) Insert shows an enlargement of inhibitor bound to ATP-pocket via direct hydrogen bonds shown as dark gray. (C) Sequences of phosphopeptides identified during MS analysis of DYRK1A. Modified residue is shown in bold and underlined.

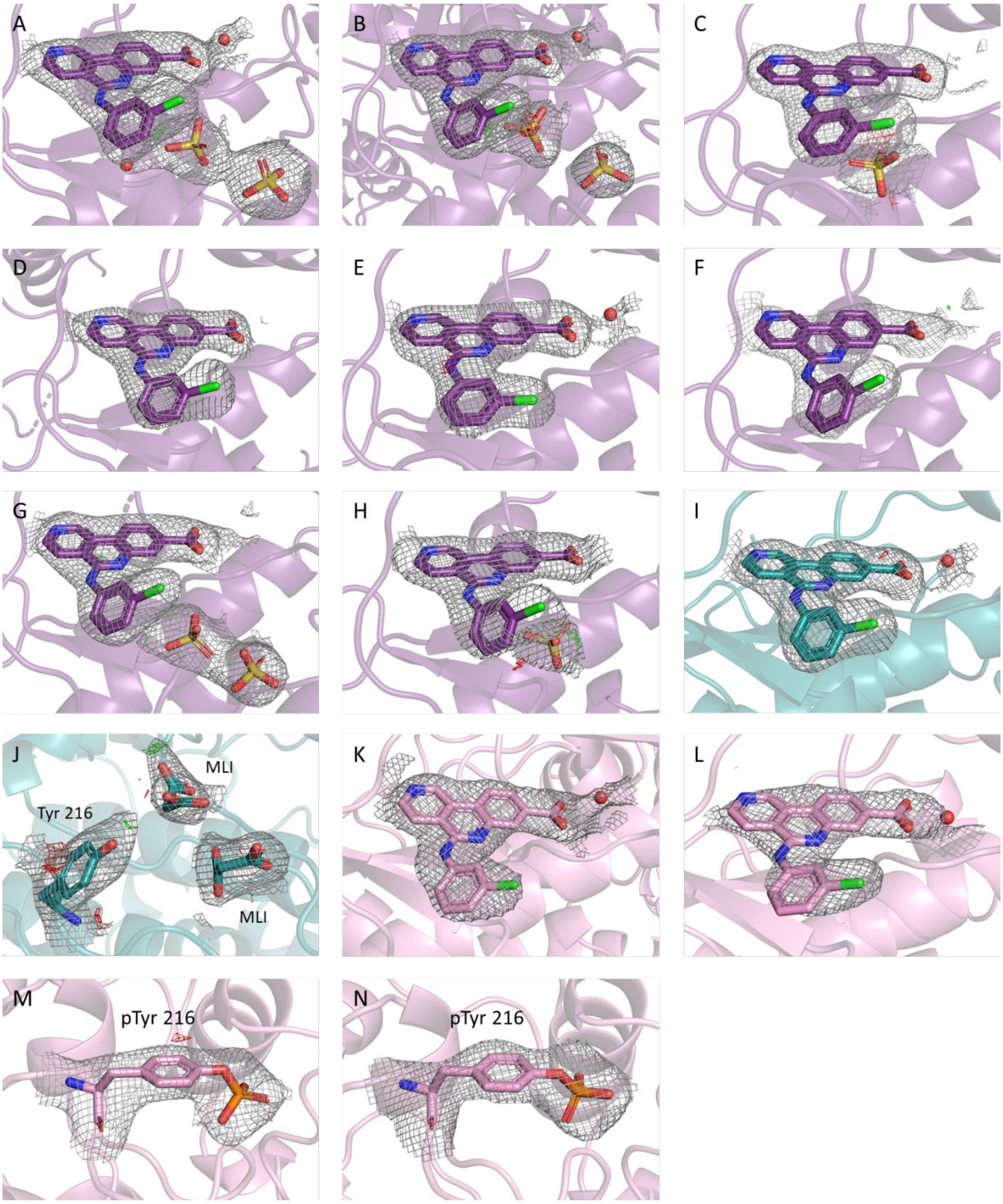

Figure S2. Electron Density map, 2Fo-fc: +1.0σ (gray); Fo-Fc omit-map: +3.0σ (green); Fo-Fc omit-map: -3.0σ (red). (A - H) CX-4945 and sulphate ions in DYRK1A/CX-4945 crystal structure for molecules A-H found in asymmetric unit, respectively, (I) CX-4945 in non-phosphorylated (Tyr216) GSK3β/CX-4945 for molecule A found in asymmetric unit, (J) Tyr 216 and malonate in non-phosphorylated (Tyr216) GSK3β/CX-4945 for molecule A found in asymmetric unit, (K, L) CX-4945 in phosphorylated GSK3β/CX-4945 crystal structure for molecules A and B found in asymmetric unit, respectively, (M, N) phosphorylated Tyr 216 in phosphorylated GSK3β/CX-4945 crystal structure for molecule A and B in asymmetric unit, respectively.

Table S2. Data collection and refinement statistics.

	**DYRK1A**	**(pTYR216) GSK3β**	**(nonpTYR216) GSK3β**
**PDB ID**	7Z5N	7Z1F	7Z1G
**Wavelength**	0.918400	0.885603	0.918400
**Resolution range**	48.11 - 2.77 (2.82 - 2.77)	46.46 - 3.0 (3.18 - 3.0)	44.14 - 2.85 (3.00 - 2.85)
**Space group**	C 1 2 1	P 31 2 1	P 43 21 2
**Unit cell**	247.682 134.317 121.717 90 96.484 90	86.001 86.001 237.735 90 90 120	84.744 84.744 195.802 90 90 90
**Total reflections**	385861 (19657)	130705 (21416)	193069 (28959)
**Unique reflections**	100435 (4979)	21174 (3368)	17303 (2484)
**Multiplicity**	3.8 (3.9)	6.2 (6.4)	11.2 (11.7)
**Completeness (%)**	99.64 (99.81)	99.24 (99.66)	98.74 (99.29)
**Mean I/sigma(I)**	10.5 (1.27)	6.0 (1.65)	14.7 (1.48)
**Wilson B-factor**	71.87	77.31	73.64
**R-merge**	0.087 (1.436)	0.189 (1.325)	0.135 (1.858)
**CC1/2**	0.997 (0.340)	0.986 (0.604)	0.996 (0.581)
**R-work**	0.2050	0.2227	0.2023
**R-free**	0.2517	0.2487	0.2282
**Number of atoms**	21926	5209	2909
**Macromolecules**	21206	5097	2751
**Ligands**	514	59	70
**Water**	206	53	88
**Protein residues**	2697	678	356
**RMS(bonds)**	0.004	0.005	0.004
**RMS(angles)**	0.65	0.83	0.81
**Ramachandran favored (%)**	94.61	94.53	95.17
**Ramachandran allowed (%)**	5.20	5.32	4.83
**Ramachandran outliers (%)**	0.19	0.15	0.00
**Rotamer outliers (%)**	1.59	1.18	2.06
**Clashscore**	5.90	4.31	1.81
**Average B-factor**	84.93	84.96	79.70
**macromolecules**	85.16	85.06	79.83
**ligands**	82.10	84.16	85.23
**solvent**	67.82	76.27	71.02

*Data for the highest resolution shell are shown in parentheses.

S3 – Dye-based Thermal Shift Assay

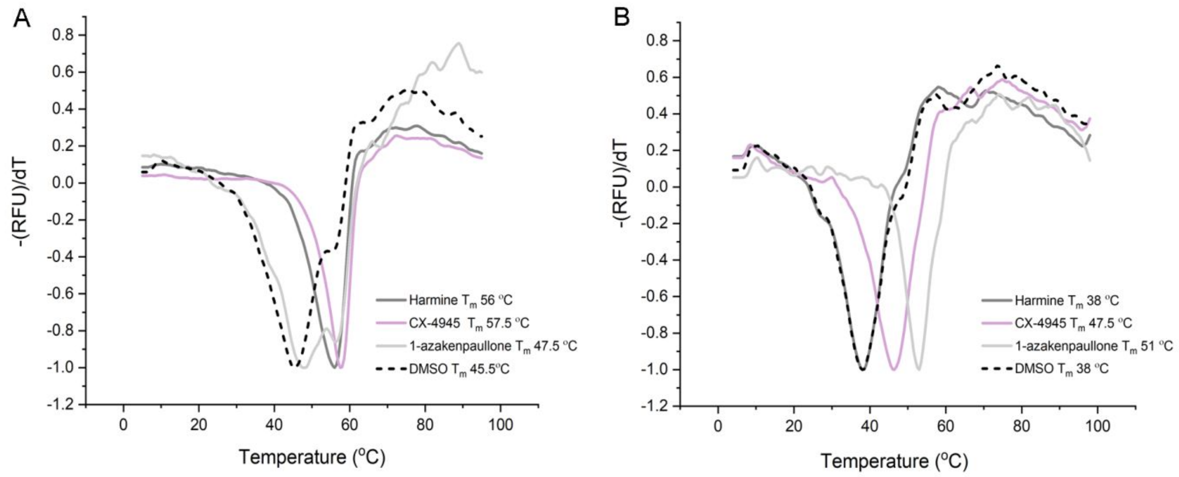

Figure S3. Thermal denaturation curves (first derivative) of apo- and inhibitor-bound DYRK1A (A) and GSK3β (B) determined in Sypro Orange dye monitored Thermal Shift Assay.

S4 – Quantum Chemistry on Small Protein-Ligand Complexes

Below we collect data related to the quantum chemical calculations on small protein-ligand complexes. The procedure we adopted was similar in both cases: 1) we extracted from the experimental structure a protein-ligand network (of interactions); 2) we capped the cut protein with protons to fulfill the octet rule and electroneutrality; 3) we ran in-pocket optimization with unconstrained optimization of proton coordinates; 4) we further reduced the size of these complexes to extract the best possible approximation to each ligand-residue interaction. Further modifications of the protein for other tests were accomplished using the respective chemical modifications followed by in-pocket optimization.

Table S3 summarizes data for DYRK1A, whereas the data for GSK3β is provided in table S4. When estimating interaction energies between the inhibitor and a functional group, one may try to isolate as much as possible each functionality or alternatively we include macrosystems, which may extend over more than one amino acid. Inclusion of larger systems contains neighbourhood and chemical environment effects, but it also adds extra interactions. For instance, full inclusion of a cysteine residue also includes dispersion forces from the sulphur atom. Because in protein biochemistry the building block is the amino acid, we tried respecting this as much as possible and only in exceptional situations we further partitioned the system. Further, the chemical environment is included for the building block.

We start by comparing the Lysin interactions between the two proteins, which seem contradictory: the ligand-lysin interaction in DYRK1A is stronger, though the hydrogen bond is longer. Reducing the system to the absolute minimum, *i.e.*, converting the lysin into methylammonium, leads to interactions of the same magnitude. We conclude therefore the difference in interaction energies lies in the interactions with the lysins' side chains. Using this information, we estimate the CX-4945-lysin interaction to be -11.273 kcal/mol hydrogen bond and ionic bridge and -1.320 kcal/mol interactions with the side chain. The calculation with

charge neutral lysin allows us to estimate the strength of the ionic bridge interaction, which we estimate at -7.315 kcal/mol. Note that the difference between DYRK1A and GSK3β lies in the side chain interactions, so the contributions of hydrogen-bond and ionic bridge are identical.

Similar procedures were applied on the other groups involved in hydrogen bonding. To estimate the hydrogen bond interaction between the carboxylate and Phe308 we replaced the amide with a carbonyl, *i.e.*, we transformed the NH set of atoms into CH_2. Though present, the stabilization by hydrogen bonding is negligible (-0.876 kcal/mol).

Table S3. Interaction data for CX-4945 in DYRK1A. r_{AH} is the acceptor-proton distance in a hydrogen bond, AHD is the angle between acceptor, proton and donor in the same interaction. If nothing is stated in the Residue or Comment columns, the interaction is with the whole residue. Subscripted *m* states for main chain, while an *s* means side chain.

Code	ΔE (kcal/mol)	r_{AH} (Å)	AHD (°)	Residue	Comment
HB1	-12.6	1.79	158.4	Lys188	---
HB1'	-5.3	1.79	158.4	Lys188	$NH3 \rightarrow NH_2$
HB2	-13.5	1.70	163.7	Asp307	---
HB3	-9.6	1.75	164.8	Leu241	---
HB3'	-5.0	1.75	164.8	Leu241	only amide
HB4	-4.8	3.76	132.0	Phe308	---
HB4'	-3.9	3.76	132.0	Phe308	$NH \rightarrow CH_2$
HB5	-1.2	1.58	173.7	Glu203	with H_2O
HB5'	1.3	1.58	173.7	Glu203	no H_2O
***π*S1**	-11.6	---	---	Glu239, Leu241, Ser242	Has H-bond
***π*S2**	-2.1	---	---	Glu239	---
***π*S3**	-1.7	---	---	Ser242	---
***π*SA**	-7.5	---	---	$(Gly166, Lys167, Gly168)_m$ + $(Phe170, Val173)_s$	---
LiPr1	-2.0	---	---	Val222	---
LiPr2	-2.7	---	---	Val306	---
LA	-14.8	---	---	$(Ile165, Ala186, Val173, Phe238, Met240, Leu294, Val222, Val306)_s$	---

Table S4. Interaction data for CX-4945 in GSK3β. r_{AH} is the acceptor-proton distance in a hydrogen bond, AHD is the angle between acceptor, proton and donor in the same interaction. If nothing is stated in the Residue or Comment columns, the interaction is with the whole residue. Subscripted *m* states for main chain, while an *s* means side chain.

Code	ΔE (kcal/mol)	r_{AH} (Å)	AHD (°)	Residue	Comment
HB1	-11.8	1.50	158.5	Lys85	---
HB1'	-7.7	1.50	158.5	Lys85	with Glu97
HB2	-14.7	1.83	149.9	Asp200	---
HB3	-7.8	2.04	168.7	Val135	---

HB3'	-4.3	2.04	168.7	Val135	only amide
HB4	-13.2			Complex	
HB5	-10.8	1.50	156	Lys85	---
HB6	12.6	2.05	94.6	Glu97	with H_2O
HB7	-9.0			Complex	
HB8	-13.1	1.81	151.5	Asp200	---
HB9	-11.7	1.81	151.5	Asp200	No CO_2
**π**SA	-9.1	---	---	Asp133, Val135	---
LiPr	-1.2	---	---	Val110	---
LA	-15.7			(Ile62, Ala83, Val70, Leu132, Tyr134, Leu188, Val110, Cys199)$_s$	---

The π-stacking interactions described in the tables above involve, as usual, interactions with full residues. This means that the whole π-stacking stabilization is furthermore affected by hydrogen bond contributions. These must be removed, according to what we calculated. Subtracting the stabilization estimated using only the amide leads to the values reported in the main text. For the explicitly lipophilic interactions we used only main and side chain parts of the amino acids. Therefore, such corrections are no longer needed.
Calculation files for the data we show here, also containing geometries, is provided separately in the supplementary material.

S5 – Interaction Maps for CX-4945 in different proteins

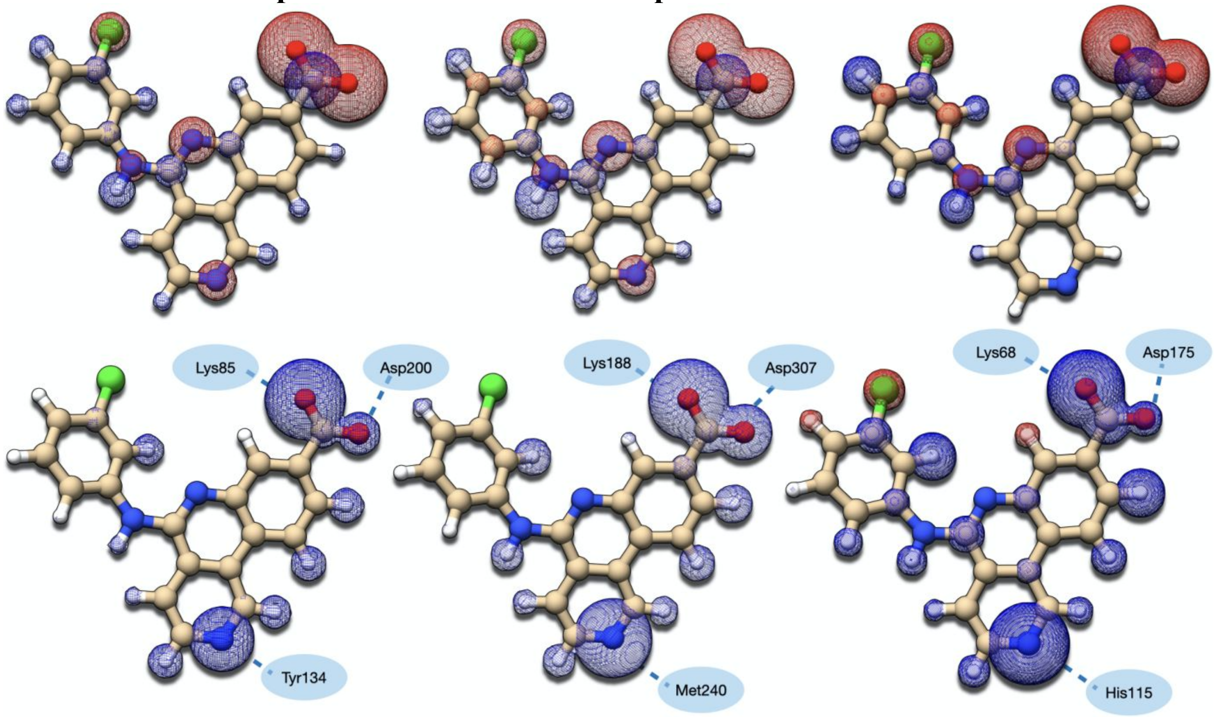

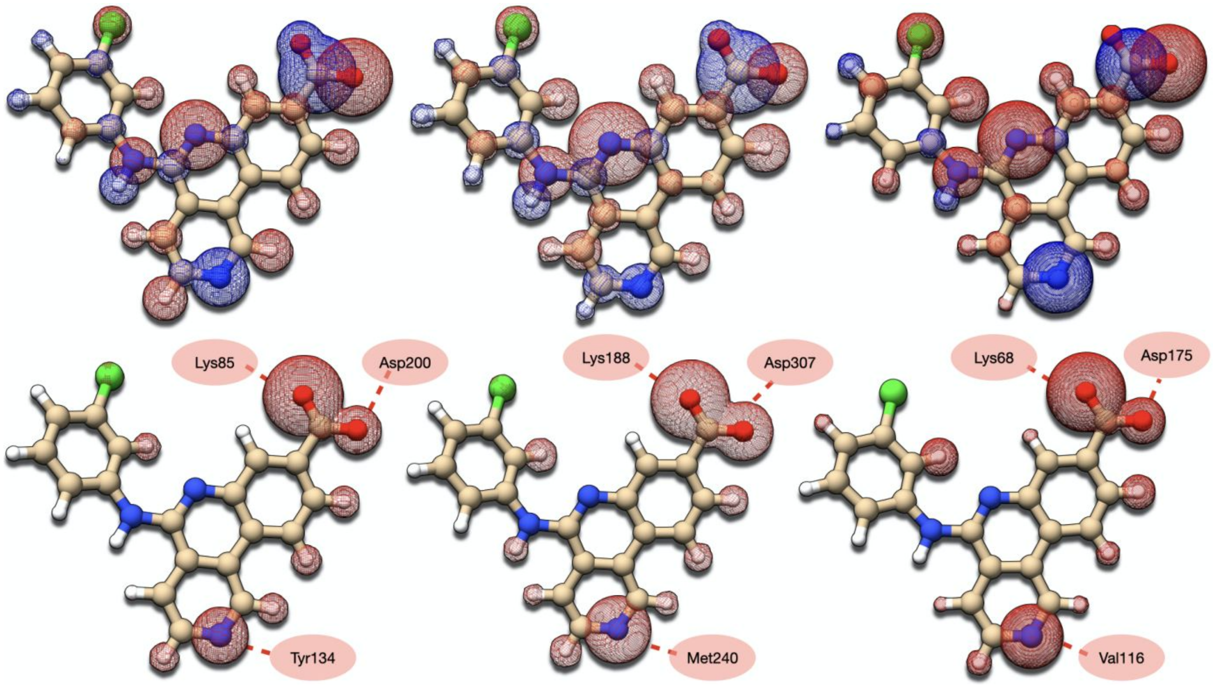

Figure S4. Interaction maps for solvation, then exchange-polarization, charge transfer and finally overlap-repulsion. Much like in the main text, we have on the left GSK3β, then DYRK1A, then CK2α.

The calculated polarization terms reflect the electrostatic environment that the proteins provide to the ligand. By definition,[3] polarization is the deformation of a molecule's electronic density caused by the charge distribution of its interacting partner when binding takes place. Interestingly, there are only minor differences in the polarizing effect/electrostatic environment of GSK3β and CK2α. The total polarization energy is significantly lower in the case of DYRK1A, which attests for its weaker electrostatic power concluded above.

Exchange-Polarization and repulsion maps show where protein and ligand are closer. These are primarily the atoms involved in hydrogen bonding and to minor extent some protons from the benzo-naphthyridine group. In the case of DYRK1A, the repulsion maps show furthermore a larger interaction sphere around the oxygen with the Asp contact (atom OD2). This is caused by the aromatic ring on Phe238. CK2α also points a phenyl group to the carboxylate in a similar fashion (Phe113), though the distance between carboxylate and aromatic ring is larger here. Repulsion maps show further differences on nitrogen number 8, resulting from the relative size of the electronic clouds of a phenol ring (Tyr134) against those of a sulfur atom (Met240 in DYRK1A and Met163 in CK2α). Though DYRK1A and CK2α share many elements in their respective pockets, repulsion is weaker in the latter by almost 13 kcal/mol. This reflects how well the ligand sits inside the protein's pocket.

The charge transfer behavior of the ligand's carboxylate seems to be somewhat conserved in all kinases, though the maps hint that such an interaction is stronger in DYRK1A than it is in GSK3β. This is meaningful given the relative electrostatic-lipophilic character of the two proteins. We note that charge transfer brings electrons from where they over accumulate to where they are lacking, i.e., it consists of the flow of electrons between two molecules or

fragments. Therefore, charge transfer weakens electrostatics, accounting for the values calculated for DYRK1A and GSK3β. But the carboxylate group alone is unable to account for the overall charge transfer stabilization of CK2α. For this, we invoke the nitrogen atom in ring C. Because this nitrogen is involved in a strong hydrogen bond interaction with the protein, our conclusion is that this interaction has a strong covalent character. Again, this corroborates the relative lipophilic character between CK2α and GSK3β. Interestingly, the proton-acceptor distance in the hydrogen bond between the nitrogen atom in ring C and the main chain amide from the protein is shorter for DYRK1A than it is for CK2α. Nevertheless, the acceptor-proton-donor angle seems to be more favorable in CK2α, which could account for the results we report. We therefore consider cautiously the fact that also charge transfer reproduces qualitatively the order of the experimental protein-ligand affinities. We note furthermore that the charge transfer effect correlates well with the relative electrostatic-lipophilic characters of the different proteins. This means that charge transfer may also well be a consequence and not a cause.

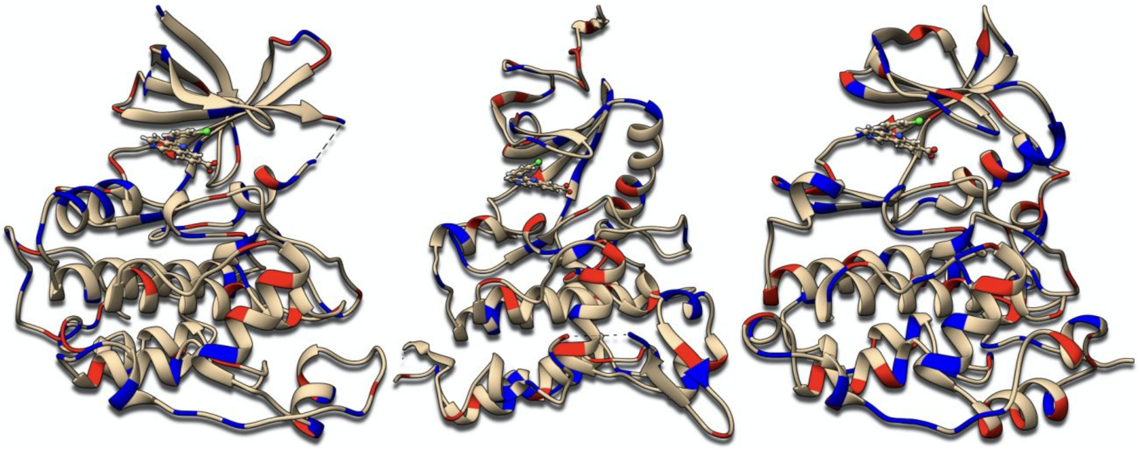

Figure S5. The distribution of positively and negatively charged amino acids over the three proteins. Pictures ordered as GSK3β, then DYRK1A, then CK2α.

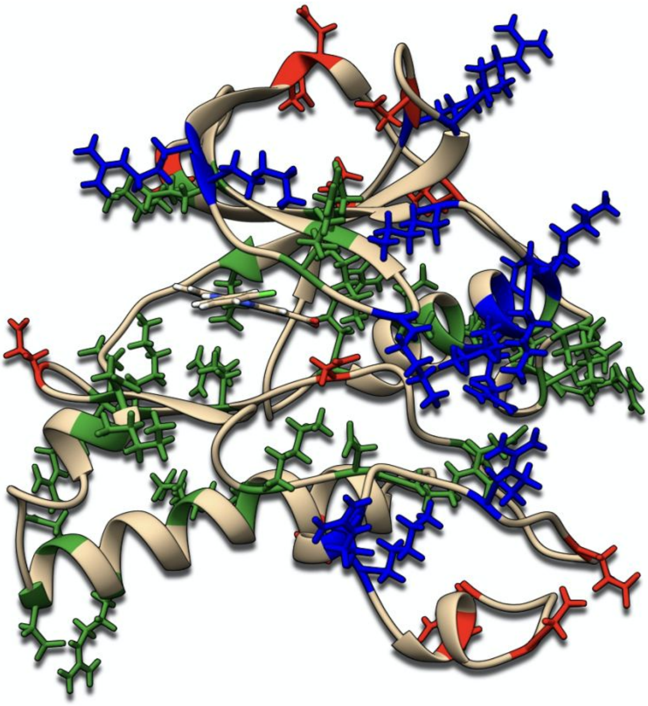

Figure S6. Formal charge cancellation on the structure of CK2α. Amino acids with charge formally cancelled by another amino acid in the vicinity are marked in green. Amino acids marked with blue are positively charged and their charges are not cancelled by their neighbors, and amino acids marked in red are negatively charged.

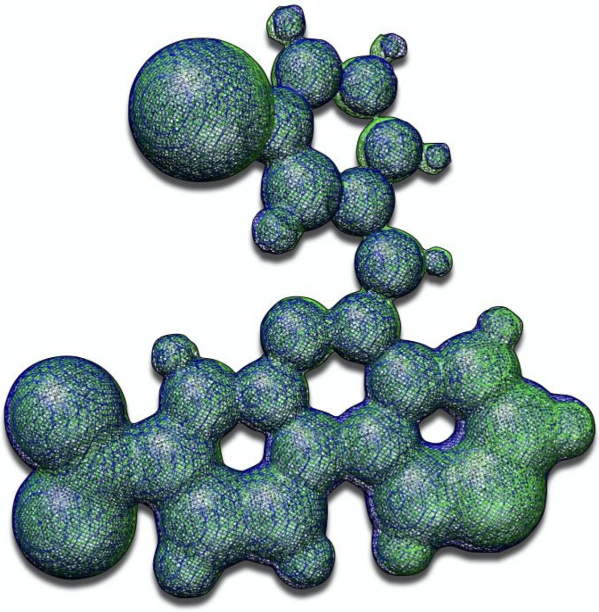

Figure S7. Overlay of dispersion maps for DYRK1A (green) and GSK3β (blue). All apparent differences were traced to fine subtleties in the binding poses.

Table S5. Quantitative energy decomposition data for the three protein-ligand complexes studied in this work. Values in kcal/mol.

	GSK3β	DYRK1A	CK2α
E_{ES}	-153.1	-124.0	-147.5
E_{POL}	-35.4	-21.5	-34.2
E_{CT}	-3.3	-6.3	-12.0
E_{REP}	55.4	65.4	52.6
E_{DISP}	-33.0	-39.8	-41.7
E_{SOLV}	126.8	80.9	134.1
E_{INT}	-42.6	-45.3	-48.7

Table S6. Strength of the lipophilic interactions between ligand and protein split over different fragments of the ligand. Phenyl corresponds to the aromatic carbons of the chloro-phenyl group, benzo-naphthyridine the aromatic carbons of this group and C_{ar} is the total contribution from aromatic carbons. Other contributions are atom specific. The last contribution is from the carboxylate. Note that these values do not contain "self-dispersion interactions", i.e., the dispersion forces resulting from atoms within the same molecule. Consequently, the values in this table do not sum up to the values in the previous table.

Group	GSK3β	DYRK1A	CK2α
$Phenyl$	-4.3	-5.5	-7.8
$benzo-naphthyridine$	-16.0	-18.3	-18.4
C_{ar}	-20.3	-23.8	-26.2

Cl	-2.9	-3.8	-3.4
N	-3.9	-4.6	-4.8
H	-3.6	-4.4	-4.6
CO_2^-	-6.3	-7.7	-7.1

Table S7. Reduced form of the previous table, *i.e.*, we divide each entry of the previous table by the number of atoms in each group. The latter is provided in parenthesis.

Group	GSK3β	DYRK1A	CK2α
$Phenyl$ (6)	-0.7	-0.9	-1.3
$benzo-naphthyridine$ (12)	-1.3	-1.5	-1.5
C_{ar} (18)	-1.1	-1.3	-1.5
Cl (1)	-2.9	-3.8	-3.4
N (3)	-1.3	-1.5	-1.6
H (11)	-0.3	-0.4	-0.4
CO_2^- (3)	-2.1	-2.6	-2.4

Table S8. Strength of dispersion interactions between the chloro-phenyl fragment of the ligand and specific (parts of) amino acids from the pocket. If nothing is stated in the Residue column, then the interaction is estimated using only the side chain. Subscripted *m* states that only main chains were used in the calculation.

Protein	ΔE (kcal/mol)	Residue
CK2α	-2.2	His160
	-2.4	Leu45
	-3.5	$(\text{Arg47, Gly46, Leu45})_m$
	-1.8	Val53
	-10.0	sum
DYRK1A	-0.5	Asn244
	-1.7	Ile165
	-4.1	$(\text{Ile165, Gly166, Lys167})_m$
	-1.4	Phe170
	-7.7	sum
GSK3β	-1.3	Ile62
	-3.0	$(\text{Ile62, Gly63})_m$
	-1.2	Phe67
	-1.8	Val70
	-7.2	sum

S6 – Overall Balance in Quantum Mechanical Calculations

General considerations regarding choice of method:

When running energy decomposition calculations on large pocket-ligand systems (with over 3500 atoms), computationally efficient quantum chemical methods are required. This is

precisely the strength of our newly developed energy decomposition analysis, which is compatible with semi-empirical Hamiltonians.

In a recent publication,[4] Villot and coworkers verified that GFN2-xTB may present limitations when evaluating absolute binding energies. This also matches our own experience, where, *e.g.*, in an analysis of the S66x10 database[5] restricted to van der Waals pairs of biological interest, we observed that GFN2xTB's performance is lower than PM6-D3H4[6].[3] However, this concerns primarily the calculation of absolute binding energies. In this work, focus is given to the calculation of relative contributions. Jorgensen and coworkers have shown on several occasions that though semi-empirical methods may lack accuracy for obtaining absolute energetics, relative ones may be obtained quite accurately.[7] Similar observations are made for GFN2-xTB in our studies.[6]

Since we are interested in relative binding energies, it is important to consider:

i) A good dispersion model. From the series of dispersion corrections compatible with semi-empirical methods, data suggests that D4 is the most robust and reliable method.[8,9]

ii) A good and robust electrostatic model as provided in general by DFTB methods.[8] This is particularly important when treating hydrogen-bonds, so that additional and separate corrections are not required (as in PM6-D3H4X). Note that technically these corrections are unproblematic. However, their assignment to specific forces in an energy decomposition analysis is highly questionable.

iii) A robust solvation model, as is the case of ALPB.

Based on all these considerations, the combination of GFN2-xTB with ALPB came as the natural choice for the calculations as the best compromise between (relative) accuracy and efficiency.

Further confirmation was obtained with tests performed on the systems studied in this work. We observed for instance that smaller pockets did not lead to results in agreement with the experimental data: smaller GSK3β pockets miss the electrostatic environment offered by the protein and the binding energy, relative to DYRK1A, is incorrect. The same applies for CK2α, which furthermore required a significant effort to cut a balanced protein pocket (see more details below). This brings us then to the last point. Though our calculations rely on error cancelation, like any other computational technique, the results we present reflect, within the approximations employed, the experimental data collected.

Considerations regarding pocket size:

To evaluate the stability and meaningfulness of our calculations we repeated the calculations on each protein using different pocket sizes.

In the case of GSK3β and DYRK1A the situation was straightforward. Small pockets with approximately half of the size of the large pocket simulations resulted in the wrong order of binding energies when compared to the experimental affinities. Since large pockets lead to results in agreement with the experimental data, we conclude this is to good extent an effect of long-range electrostatics, which matches the results and discussion presented in the main text. We note that we considered several different cuts for the small pockets, the results, though slightly different, were consistent.

The case of CK2α was however more complicated due to the charge distribution on the protein. Small pockets lead to results consistent with the experimental observations. Large

pockets are extremely problematic. This is particularly visible in figures S5 and S6 which show that there are in this protein regions of predominantly positively charged residues, whereas in other regions the predominance is of negatively charged amino acids. This makes the task of cutting the pocket extremely arduous while simultaneously retaining consistency, with the experimental data but also from the computational point of view. The results presented in the main section result from one of the most stable large pockets we performed calculations, which was furthermore consistent with the small pocket calculations. The delimitation of the pocket required structural considerations and careful design, in both cases to ensure the consistency with respect to the global protonation state returned from MAESTRO.

We note that though the small pocket calculations do not reflect the correct order for binding affinities, the conclusions from the main manuscript remain unaffected even in that case. This reflects the care that must be taken when cutting protein pockets due to imbalanced electrostatics that might not be visible when calculating quantum chemical interaction energies using the supermolecular approach. We note furthermore that when using our energy decomposition analysis, the charge distribution on the protein has impact at many levels. It impairs directly the electrostatics, solvation and polarization energies, but also indirectly charge transfer. Data on other calculations and the geometries used for the calculations are provided separately in this supplementary material.

Static vs. Dynamic:

Simulation work on protein-ligand complexes typically involves extensive dynamical simulations to increase conformational sampling. This is an essential component to build and understand robustly and reliably entropy effects, thus also Gibbs free energies, from *in silico* models. However, in this work, sampling methods were not employed. Figures S1 and 5 (main manuscript) provide us with support for neglecting entropy and sampling effects from the models herein built. As the overlays show, the binding poses are remarkably conserved over the different kinases (Figure 5). Even within the asymmetric unit of the crystal of one protein-ligand complex (Figure S1), binding poses are all alike. This indicates that conformational aspects in the bound complexes are of lesser importance, and it allows us to focus exclusively in improving the binding enthalpy contributions. This is where the energy decomposition analysis proves critical because it permits one to understand the nature of the interactions between two molecules. One could however still argue that small conformational sampling effects could potentially influence the calculated enthalpies (or more precisely binding energies). This goes however against our observations.[6] Indeed, absolute contributions of different forces may vary with conformation, however, the essence of the interactions (maps) remains largely unaffected.

Checking the validity of GFN2-xTB for current application:

Despite our confidence in the validity of using GFN2-xTB/ALPB for calculating relative contributions to interaction energies, we feel that the message of the current manuscript will be strengthened by corroborating some of calculations shown in the main text. To this end, we decided to investigate the validity of our claims regarding the interaction between His160 and CX-4945 in the complex with CK2α. Starting from the in-pocket optimized structures of the complexes, DLPNO-CCSD(T)/def2-TZVP calculations were run to determine the

(DLPNO-)CCSD and (DLPNO-)CCSD(T) gas phase binding energies. We also evaluated the contribution from the solvation model by including calculations using the COSMO model (based on PM6-D3H4 calculations). Table S9 summarizes our findings.

Table S9. Strength of the T-stack and hydrogen-bond interactions relevant to evaluate the interaction between His160 and the chloro-phenyl moiety of the ligand. *Ab initio* calculations performed using the basis set mentioned in text of this supplementary material. Binding energies evaluated in the vacuum (gas) and with ALPB.

		Binding Energy (kcal/mol)	
		gas	ALPB
CCSD	T-stack	-3.47	-2.80
	H-bond	-6.56	-2.26
CCSD(T)	T-stack	-3.55	-2.88
	H-bond	-6.67	-2.37
GFN2-xTB	T-stack	-2.86	-2.18
	H-bond	-5.26	-0.96
PM6-D3H4X	T-stack	-3.07	-2.40
	H-bond	-8.40	-4.10

As evidenced by the data, GFN2-xTB results are a close match to the higher-level calculations, both CCSD and CCSD(T), which themselves are also quite close to one another. Though there is a larger deviation in the strength of the hydrogen-bond in the gas phase, results are still superior than with PM6-D3H4X. In conclusion, the deviations at the GFN2-xTB level are all within the expectable.

Having determined the validity of GFN2-xTB to evaluate the strength of the 2 interactions at stake, we proceeded to include the effect of the solvation model. After all, a close inspection of table S9 reveals that the hydrogen-bond is actually stronger than the T-stack in the absence of the solvation model. This is in contradiction with our claims in the main manuscript. We observe that solvation impacts significantly more the hydrogen-bond contact than it does the T-stack. The higher-level calculations corroborate nonetheless the GFN2-xTB/ALPB data. Though the difference in energy is less pronounced, one still expects the T-stack to dominate over the hydrogen bond.

The strength of a T-stack:

We also performed a potential energy scan for the two aromatic fragments involved the chloro-phenyl and the imidazole rings. Here we kept the distance between both fragments fixed at the value from the crystal structure, and we positioned them at different relative orientations. For each relative orientation, the interaction energy between the two molecules was recorded. We observed that the optimal interaction is about -2.7 kcal/mol, which is, given the distance restrain imposed, the strongest the interaction can get. This is remarkably close to the value we calculated for the crystal of CK2α, which was furthermore obtained from non-static molecules, *i.e.*, for conditions in which entropic and vibrational effects contribute with deviations from the optimal orientation. Further, due to the influence of the water mediated hydrogen bond, shifts in the optimal binding pose may occur. From our energy scan calculations, we then conclude

that the orientation of His160 in the crystal is particularly suitable to promote the T-shaped interaction between the 2 aromatic rings. Finally, full optimization of the T-stack interaction between the two aromatic rings (without any constraint whatsoever) showed that the best possible interaction energy for imidazole T-stacked to the chloro-phenyl ring is -2.93 kcal/mol, again quite close to the value from the crystal (energetically and structurally). This reinforces even more our conclusions. The calculations indicate that CK2α's picomolar affinity to CX-4945 is related to a histidine residue exclusive to this kinase and the quasi-optimal orientation this residue's side chain adopts to interact with the inhibitor in an aromatic T-stacked interaction. Hydrogen bonding, with nitrogen 13 is expected to play a secondary role for selectivity (but not for affinity).

References

1) Birus, R.; El-Awaad, E.; Ballentin, L.; Alchab, F.; Aichele, D.; Ettouati, L.; Goetz, C.; Le Borgne, M.; Jose, J. 4,5,7-Trisubstituted indeno[1,2-b]indole inhibits CK2 activity in tumor cells equivalent to CX-4945 and shows strong anti-migratory effects. *FEBS Open Bio* **2022**, *12*, 394.

2) Ertl, P.; Rohde, B.; Selzer, P. Fast Calculation of Molecular Polar Surface Area as a Sum of Fragment-Based Contributions and Its Application to the Prediction of Drug Transport Properties. *J. Med. Chem.* **2000**, *43*, 3714.

3) Menezes, F.; Popowicz, G. M. Understanding the Nature of Interactions in Macromolecules. Manuscript Submitted 2022.

4) Villot, C.; Ballesteros, F.; Wang, D.; Lao, K. U. Coupled Cluster Benchmarking of Large Noncovalent Complexes in L7 and S12L as Well as the C60 Dimer, DNA–Ellipticine, and HIV–Indinavir. *J. Phys. Chem. A* **2022**, *126*, 4326.

5) Brauer, B.; Kesharwani, M. K.; Kozuch, S.; Martin, J. M. L. The S66x8 benchmark for noncovalent interactions revisited: explicitly correlated ab initio methods and density functional theory. *Phys. Chem. Chem. Phys.* **2016**, *18*, 20905.

6) a) Stewart, J. J. P. Optimization of parameters for semiempirical methods V: Modification of NDDO approximations and application to 70 elements. *J. Mol. Model.* **2007**, *13*, 1173. b) Grimme, S.; Antony, J.; Ehrlich, S.; Krieg, H. A consistent and accurate ab initio parametrization of density functional dispersion correction (DFT-D) for the 94 elements H-Pu. *J. Chem. Phys.* **2010**, *132*, 154104. c) Řezáč, J.; Hobza, P. Advanced corrections of hydrogen bonding and dispersion for semiempirical quantum mechanical methods. *J. Chem. Theory Comp.* **2012**, *8*, 141

7) a) Acevedo, O.; Jorgensen, W. L. Quantum and molecular mechanical Monte Carlo techniques for modeling condensed-phase reactions. *WIREs Comp. Mol. Sci.* **2014**, *4*, 422. b) Tubert-Brohman, I.; Acevedo, O.; Jorgensen, W. L. Elucidation of Hydrolysis Mechanisms for Fatty Acid Amide Hydrolase and Its Lys142Ala Variant via QM/MM Simulations. *J. Am. Chem. Soc.* **2006**, *128*, 16904. c) Guimarães, C. R. W.; Repasky, M. P.; Chandrasekhar, J.; Tirado-Rives, J.; Jorgensen, W. L. Contributions of conformational compression and preferential transition state stabilization to the rate

enhancement by chorismate mutase. *J. Am. Chem. Soc.* **2003**, *125*, 6892. d) Acevedo, O.; Jorgensen, W. L. Influence of Inter- and Intramolecular Hydrogen Bonding on Kemp Decarboxylations from QM/MM Simulations. *J. Am. Chem. Soc.* **2005**, *127*, 8829. e) Repasky, M. P.; Guimarães, C. R. W.; Chandrasekhar, J.; Tirado-Rives, J.; Jorgensen, W. L. Investigation of Solvent Effects for the Claisen Rearrangement of Chorismate to Prephenate: Mechanistic Interpretation via Near Attack Conformations. *J. Am. Chem. Soc.* **2003**, *125*, 6663.

8) Grimme, S.; Bannwarth, C.; Caldeweyher, E.; Pisarek, J.; Hansen, A. A general intermolecular force field based on tight-binding quantum chemical calculations featured. *J. Chem. Phys.* **2017**, *147*, 161708.

9) Caldeweyher, E.; Ehlert, S.; Hansen, A.; Neugebauer, H.; Spicher, S.; Bannwarth, C.; Grimme, S. A generally applicable atomic-charge dependent London dispersion correction. *J. Chem. Phys.* **2019**, *150*, 154122.
